# Genomic analysis of oceanic cyanobacterial myoviruses compared with T4-like myoviruses from diverse hosts and environments

**DOI:** 10.1111/j.1462-2920.2010.02280.x

**Published:** 2010-11

**Authors:** Matthew B Sullivan, Katherine H Huang, Julio C Ignacio-Espinoza, Aaron M Berlin, Libusha Kelly, Peter R Weigele, Alicia S DeFrancesco, Suzanne E Kern, Luke R Thompson, Sarah Young, Chandri Yandava, Ross Fu, Bryan Krastins, Michael Chase, David Sarracino, Marcia S Osburne, Matthew R Henn, Sallie W Chisholm

**Affiliations:** 1 Massachusetts Institute of TechnologyCambridge, MA, USA; 2 University of ArizonaTucson, AZ, USA; 3 Broad InstituteCambridge, MA, USA; 4 New England Biolabs, Chemical Biology Division240 County Road, Ipswich, MA 01938, USA; 5 Harvard PartnersCambridge, MA 02139, USA

## Abstract

T4-like myoviruses are ubiquitous, and their genes are among the most abundant documented in ocean systems. Here we compare 26 T4-like genomes, including 10 from non-cyanobacterial myoviruses, and 16 from marine cyanobacterial myoviruses (cyanophages) isolated on diverse *Prochlorococcus* or *Synechococcus* hosts. A core genome of 38 virion construction and DNA replication genes was observed in all 26 genomes, with 32 and 25 additional genes shared among the non-cyanophage and cyanophage subsets, respectively. These hierarchical cores are highly syntenic across the genomes, and sampled to saturation. The 25 cyanophage core genes include six previously described genes with putative functions (*psbA*, *mazG*, *phoH*, *hsp20*, *hli03*, *cobS*), a hypothetical protein with a potential phytanoyl-CoA dioxygenase domain, two virion structural genes, and 16 hypothetical genes. Beyond previously described cyanophage-encoded photosynthesis and phosphate stress genes, we observed core genes that may play a role in nitrogen metabolism during infection through modulation of 2-oxoglutarate. Patterns among non-core genes that may drive niche diversification revealed that phosphorus-related gene content reflects source waters rather than host strain used for isolation, and that carbon metabolism genes appear associated with putative mobile elements. As well, phages isolated on *Synechococcus* had higher genome-wide %G+C and often contained different gene subsets (e.g. *petE*, *zwf*, *gnd*, *prnA*, *cpeT*) than those isolated on *Prochlorococcus*. However, no clear diagnostic genes emerged to distinguish these phage groups, suggesting blurred boundaries possibly due to cross-infection. Finally, genome-wide comparisons of both diverse and closely related, co-isolated genomes provide a locus-to-locus variability metric that will prove valuable for interpreting metagenomic data sets.

## Introduction

### T4-like phages

Double-stranded DNA bacteriophages (*Caudovirales*) are the primary viral types observed in marine systems. Myoviruses (contractile-tailed phages) predominate among these, as determined by viral metagenomic surveys (Breitbart *et al*., 2002; 2004; Angly *et al*., 2006; Williamson *et al*., 2008) and in culture experiments (Suttle and Chan, 1993; Waterbury and Valois, 1993; Wilson *et al*., 1993; Lu *et al*., 2001; Marston and Sallee, 2003; Sullivan *et al*., 2003). Myoviruses also dominated the viral signal in *microbial-fraction* metagenomic data sets from Hawaii (DeLong *et al*., 2006) and from the surface waters sampled in the Global Ocean Survey (GOS; Rusch *et al*., 2007; Yooseph *et al*., 2007), the latter of which reports that five of the six most abundant GOS proteins were attributed to T4-like myoviruses (Yooseph *et al*., 2007). The viral signal in these microbial metagenomes is thought to represent infecting viruses captured inside infected host cells, suggesting that T4-like phages are both numerically abundant and actively infectious (DeLong *et al*., 2006).

The canonical *Escherichia coli* bacteriophageT4 has a well-characterized infection cycle, genome and transcriptome (Luke *et al*., 2002; Miller *et al*., 2003a). A watershed of papers has defined the ‘core’ genes representative of the growing family of known T4-like phages. Relatively early work (Hambly *et al*., 2001) first noted that the ocean cyanobacterial T4-like virus S-PM2 had a module of capsid gene sequences similar to those of phage T4 – isolated using *E. coli* from sewage – suggesting that at least portions of these phage genomes might be shared across distantly related phages. Subsequent work (Desplats *et al*., 2002) expanded these observations, using a larger fraction of an *E. coli* T4-like phage genome (RB49) to show that the general virion structural components *and* the DNA replication apparatus were also conserved across T4-like phages. Whole-genome comparison followed that compared the archetype T4 phage with marine T4-like vibriophage KVP40 (Miller *et al*., 2003b), and T4-like coliphage JS98 (Chibani-Chennoufi *et al*., 2004); these studies showed that the ‘T4 core’ genes encode structural proteins to produce virus particles, as well as the metabolic machinery required for infection of the host.

As new genomes became available, further whole-genome comparisons refined our understanding of the T4 core (e.g. phages T4, RB49 and Aeh1 share 90 genes, Comeau *et al*., 2007) and shifted the focus to characterizing the flexible genome of T4-like phages (Nolan *et al*., 2006). These flexible genes encode proteins that interact with the host cell, e.g. tail fibres and internal scaffolding proteins, or likely offer other niche-defining functions such as base modification and differential complements of tRNAs (Comeau *et al*., 2007). Most of these genes are thought to represent ancient lateral transfer events, as 90% of them exhibited early/middle promoter control similar to that seen for the corresponding T4 core genes (Nolan *et al*., 2006).

### Cyanobacterial T4-like phages

Ocean microbes drive globally important biogeochemical cycles, including carbon, oxygen, nitrogen and sulfur cycles (Arrigo, 2005; Howard *et al*., 2006; Karl, 2007), and the enormous numbers of ocean viruses (typically > 10^7^ ml^−1^, or approximately 10 for every microbial cell) drive the evolution of microbial processes through host mortality (Fuhrman, 1999; Wommack and Colwell, 2000; Weinbauer, 2004; Suttle, 2005), horizontal gene transfer (Paul, 1999; Miller, 2001) and the modulation of host metabolism (Breitbart *et al*., 2007). Among marine microbes, the picocyanobacteria *Prochlorococcus* and *Synechococcus* are highly abundant (Waterbury *et al*., 1979; 1986; Partensky *et al*., 1999), and some estimates suggest that they account for as much as one-third of oceanic primary production (Li, 1994; 1995;). These two genera are commonly present at 10^5^ cells ml^−1^ and usually co-occur: *Prochlorococcus* is numerically dominant in the vast, low-nutrient open oceans (Partensky *et al*., 1999; Johnson *et al*., 2006; Coleman and Chisholm, 2007), while *Synechococcus* dominates in coastal waters (Waterbury *et al*., 1979; 1986;).

In previous studies, four *Prochlorococcus* and *Synechococcus* T4-like cyanophage genomes were found to share up to 45 genes (out of ∼150 total) with the non-cyanophages (Mann *et al*., 2005; Sullivan *et al*., 2005; Weigele *et al*., 2007, but also see Millard *et al*., 2009 and *Note added in proof*). In addition, these studies revealed the power of phage–host co-evolution in the context of ocean-basin scale ecological settings. For example, cyanophage genomes were found to contain ‘host’ genes involved in central host metabolism and photosynthesis (Mann *et al*., 2003; Lindell *et al*., 2004; Millard *et al*., 2004; 2009; Mann *et al*., 2005; Sullivan *et al*., 2005; Weigele *et al*., 2007), and these genes are expressed during phage infection (Lindell *et al*., 2005; 2007; Clokie *et al*., 2006). Further, the viral version of these host genes dominates the GOS surface ocean *microbial-fraction* metagenomes, e.g. 60% of the identifiable *psbA* genes were viral (Sharon *et al*., 2007). The distributions of these host photosynthetic genes among phage types appear driven by the physiology of the phage (e.g. host range for *psbD* and lytic cycle length for *psbA*, Sullivan *et al*., 2006). In fact cyanophages may be among the drivers of photosystem evolution as portions of the ‘host’ genes carried on cyanophages are able to recombine back into the host gene pool (Zeidner *et al*., 2005; Sullivan *et al*., 2006).

In contrast to the near-ubiquity of the core photosystem II *psbA* gene present in cyanophage genomes, other ‘host’ genes are sporadically distributed among cyanophage genomes but also may impact phage fitness. On the one hand, T4-like viral contigs assembled from marine metagenomes contain up to seven clustered photosystem I genes thought to form an intact monomeric PSI complex to funnel reducing power from electron transport chains to PSI-related functions during infection (Sharon *et al*., 2009). Interestingly, such PSI genes have yet to be identified in any genome from a cyanophage isolate (Chen *et al*., 2002; Mann *et al*., 2005; Sullivan *et al*., 2005; Weigele *et al*., 2007; Millard *et al*., 2009). On the other hand, the functional role of cyanophage-encoded phycobilin synthesis genes (*pcyA* and *pebS*) remains a mystery (Dammeyer *et al*., 2008). In this case, despite the fact that *Prochlorococcus* hosts lack intact phycobilisomes and that these cyanophage-encoded genes are highly divergent relative to host copies, they are expressed *in vivo* during infection and are functional *in vitro* (Dammeyer *et al*., 2008). It is likely that these and other sporadically distributed genes serve specific niche-defining roles for phages' adaptation to their particular hosts and environments that will reveal themselves as more genome and physiology data become available.

Here we expand the T4-like cyanophage database, nearly doubling the number of T4-like phage genomes by adding 12 new ocean cyanophage genomes to the previous four (Table 1). We use this augmented database to explore the ecology and evolution of T4-like cyanophages through an analysis of the genomes of 16 marine cyanophages compared with 10 non-cyanophage T4-like genomes from the Tulane Genome Sequencing Project (http://phage.bioc.tulane.edu/). The cyanophages were isolated from 15 different habitats over a period of 16 years, using 10 different host strains (four *Prochlorococcus* and six *Synechococcus*), while the non-cyanophages were isolated over decades using at least seven different source waters and six different hosts. Thus, these conditions optimize the potential for revealing diversity across the 26 phage isolates (Table 1) examined in this study. With this data set, we asked the following questions: What gene sets are shared and not shared among various hierarchical groupings of T4-like phages, and how do these genes inform our understanding of T4-like cyanophage and non-cyanophage biology? What mechanisms likely drive differential and sporadic distribution of non-shared genes among the cyanophages?

**Table 1:** General features of the T4-like genomes and isolates

Published name	Genbank accession #	Original host	Genome Size (kb)	# ORFs	%G + C	Source water description	Date water sampled	# tRNA	Genome publication
*Cyanophages*									
P-SSM2	AY939844	*Prochlorococcus* NATL1A	252.4	334	35.5%	Atlantic Ocean oligotrophic gyre, 100 m	6-Jun-00	1	Sullivan *et al.*^2005^
P-SSM4	AY940168	*Prochlorococcus* NATL2A	178.2	221	36.7%	Atlantic Ocean oligotrophic gyre, 10 m	6-Jun-00	0	Sullivan *et al.* 2005
P-HM1	GU071101	*Prochlorococcus* MED4	181	241	38.0%	Pacific Ocean oligotrophic gyre, 125 m	9-Mar-06	0	this study
P-HM2	GU075905	*Prochlorococcus* MED4	183.8	242	38.0%	Pacific Ocean oligotrophic gyre, 125 m	9-Mar-06	0	this study
P-RSM4	GU071099	*Prochlorococcus* MIT9303	176.4	239	38.0%	Red Sea, oligotrophic, 130 m	13-Sep-00	3	this study
P-SSM7	GU071103	*Prochlorococcus* NATL1A	182.2	237	37.0%	Atlantic Ocean oligotrophic gyre, 120 m	Sep-99	4	this study

S-PM2	AJ630128	*Synechococcus*WH7803	196.3	244	37.8%	English Channel, 0 m	23-Sep-92	25*	Mann *et al.* 2005
Syn9	DQ149023	*Synechococcus*WH8109	177.3	228	40.50%	Atlantic Ocean coastal (Woods Hole), 0 m	Oct-90	6	Weigele *et al.* 2007
Syn19	GU071106	*Synechococcus*WH8109	175.2	215	41.0%	Atlantic Ocean oligotrophic gyre, 0 m	Jul-90	6	this study
Syn33	GU071108	*Synechococcus*WH7803	174.4	227	40.0%	Atlantic Ocean (Gulf Stream), 0 m	Jan-95	5	this study
Syn1	GU071105	*Synechococcus*WH8101	191.2	234	41.0%	Atlantic Ocean coastal (Woods Hole), 0 m	Aug-90	6	this study
S-ShM2	GU071096	*Synechococcus*WH8102	179.6	230	41.0%	Atlantic Ocean coastal (continental shelf), 0 m	16-Sep-01	1	this study
S-SM2	GU071095	*Synechococcus*WH8017	190.8	267	40.0%	15 m	17-Sep-01	11	this study
S-SSM7	GU071098	*Synechococcus*WH8109	232.9	319	39.0%	Atlantic Ocean oligotrophic gyre, 70 m or 95 m	22-Sep-01	5	this study
S-SSM5	GU071097	*Synechococcus*WH8102	176.2	225	40.0%	Atlantic Ocean oligotrophic gyre, 70 m	22-Sep-01	4	this study
S-SM1	GU071094	*Synechococcus*WH6501	178.5	234	41.0%	0 m	17-Sep-01	6	this study
*Non-cyanophages*									
T4	AG158101	*E. coli*B	168.9	278	35.3%	likely from sewage see Abedon 2000	N.A.	8	Miller *et al.* 2003a
RB32	DQ904452	*E. coli*	165.9	270	35.3%	N.A.	N.A.	8	http://phage.bioc.tulane.edu/
RB43	AY967407	*E. coli*B	180.5	292	43.2%	Long Island, NY – sewage	N.A.	1	Nolan *et al.* 2006
RB49	AY343333	*E. coli*CAJ70	164	274	40.4%	Long Island, NY – sewage	N.A.	0	Nolan *et al.* 2006
RB69	AY303349	*E. coli*CAJ70	167.6	273	37.7%	Long Island, NY – sewage	N.A.	2	Nolan *et al.* 2006
KVP40	AY283928	*Vibrio parahaemolyticus*	244.8	381	42.6%	‘polluted’ coastal seawater off Japan	N.A.	24	Miller *et al.* 2003b
44RR	AY357531	*Aeromonas salmonicida*170-68	173.6	252	43.9%	Ontario Canada, Trout pond	N.A.	17	Nolan *et al.* 2006
Aeh1	AY266303	*Aeromonas hydrophila*	233.2	352	42.8%	Oshkosh, WI – treated sewage effluent	N.A.	23	Nolan *et al.* 2006
PHG25	DQ529280	*Aeromonas salmonicida*170-68	161.5	242	41.0%	Eure, France – fish hatchery	N.A.	13	Petrov *et al.* 2006
PHG31	AY962392	*Aeromonas salmonicida*95-68	172.9	247	43.9%	Ariege, France – fish hatchery	N.A.	15	Petrov *et al.* 2006

N.A. = data not available.

* One tRNA as a pseudogene.

## Results and discussion

### General features of the 16 cyanophage genomes

**Fig. 1:**
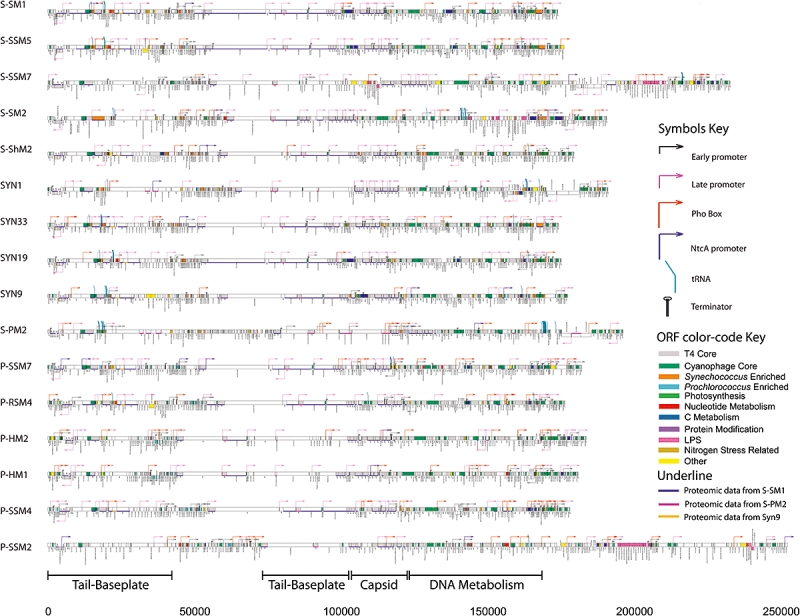
Overview of 16 cyanophage genome annotations. Each drawn box represents a predicted open reading frame (ORF) with forward strand ORFs above and reverse strand ORFs below. ORFs are colour-coded as per the legend in the figure, while colour-coded lines on the genome represent experimentally determined structural proteins (see *Experimental procedures*). For spreadsheet version of these data, please see File S1.

All available annotation information for the 16 cyanophage genomes is provided in a detailed overview figure (Fig. 1). With two exceptions, cyanophage genome sizes ranged from 174 to 196 kb (summarized in Table 1, details provided in Table S1), as commonly observed previously for non-cyanophages (Miller *et al*., 2003a,b; Nolan *et al*., 2006; Petrov *et al*., 2006). The exceptional cyanophages were S-SSM7 (232 kb) and P-SSM2 (252 kb), which contained large lipopolysaccharide gene clusters (Fig. 1, discussed below) that accounted for about 72–85% of the expanded genome size. Cyanophage genome size was correlated with the number of predicted open reading frames (ORFs) (*R*^2^ = 0.743), and there was no apparent relationship between the genome size and the genus of the host on which it was isolated (Fig. S1).

While significant variation in genome-wide %G+C exists among the non-cyanophages (Table 1), even for those isolated on the same host, we note that this metric is less variable among the cyanophage genomes (Table 1). As well, the average genome-wide %G+C content of phages isolated on *Prochlorococcus* (37.2 ± 1.0%) is significantly different (*P* ≤ 0.0001) from that of phages isolated on *Synechococcus* (40.1 ± 1.0%). Such cyanophage variability may reflect host range-constrained swapping of genetic material followed by subsequent genome-wide amelioration of the new genes in the phage genome. For example, *Synechococcus* cells have higher %G+C genomes than *Prochlorococcus* (Kettler *et al*., 2007; Dufresne *et al*., 2008) and even high %G+C material from *Synechococcus* hosts would ameliorate once in the phage genome towards the overall lower %G+C of phage genomes. In contrast, *Prochlorococcus* phage %G+Cs are often closer to that of their host genomes, so the impact of such genome-wide amelioration pressures are minimal compared with that seen in *Synechococcus*. Such observations in cyanophage-encoded core photosynthesis genes proved diagnostic for tracing intragenic recombination events among cyanophage genomes (Zeidner *et al*., 2005; Sullivan *et al*., 2006). That one cyanophage, S-PM2, deviates from the general pattern may hold clues regarding the host range of this particular phage (also see below).

### Gene distributions among hierarchical groupings of the genomes

**Fig. 2:**
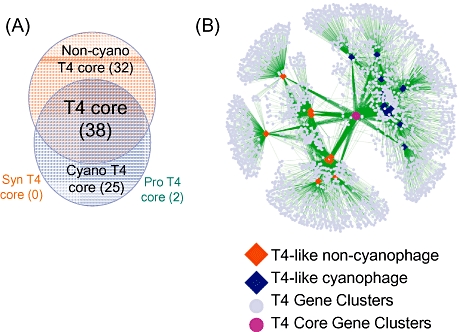
T4-like gene set relatedness representations. A. Venn diagram illustrating the hierarchical core gene sets among 26 T4-like genomes. B. T4-like phage presence/absence gene cluster network. T4 Gene Clusters (T4-GCs) were used to construct a network of phage genomes and gene clusters found in one or more of the 26 genomes. Genomes are represented as diamonds, with cyanophage genomes coloured blue and non-cyanophage coloured red. Non-core T4-GCs are represented as a light purple circle, core T4-GCs shared by all genomes are coloured dark purple. If a T4-GC is present in a phage genome, an edge (green line) is drawn between that genome and the associated T4-GC. Genomes sharing many T4-GCs are in close spatial proximity to each other in the network. A multifasta file with all ORFs examined in this study is provided to link specific ORFs, T4-GC assignments and functional annotation (File S2).

In preparation for analyses of gene content and order in the different genomes, we clustered orthologous genes into T4 Gene Clusters (‘T4-GCs’; see *Experimental procedures*), and used these to define core gene sets common to hierarchical groupings of the genomes (Fig. 2A, see discussion below). A total of 6798 predicted genes in the 26 genomes clustered into 892 T4-GCs, with 1873 genes remaining as singletons.

#### Gene presence/absence network analysis

To examine how similar the genomes are to each other with respect to the presence or absence of each T4-GC, we represented the presence/absence table as a network (Fig. 2B), which links T4-GCs to the genomes in which they are found. Genomes with many T4-GCs in common appear in close proximity due to the many connections that they share. The resulting network shows clustering of the cyanophage (blue diamonds, Fig. 2B) separate from non-cyanophage (red diamonds, Fig. 2B) T4-like genomes by this metric. Core genes shared by all 26 genomes connect the two groups of phage and are highlighted as the central purple cluster (Fig. 2B).

#### Core and pan-genomes

**Fig. 3:**
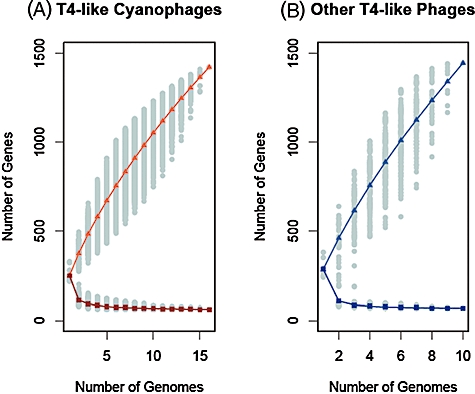
The core and pan-genomes of the (A) cyanophage and (B) non-cyanophage groups, where the core and pan-genomes are represented by square and triangles respectively. The core and pan-genomes were analysed for *k* genomes from cyanophages (*n* = 16) or non-cyanophages (*n* = 10). Each possible variation is shown as a grey point, and the line is drawn through the average. The core genome is defined as genes that are present in the selected *k* genomes. The pan-genome is the total unique genes found in *k* genomes. All variations of *n* choose *k*: *n*!/*k*!(*n* − *k*)!.

To explore the features of the core and pan-genomes of the cyanophage and non-cyanophage subsets given the number of genomes sequenced, we identified the shared and unshared gene sets of all possible combinations of choosing *k* genomes (*k* = 1 to *n*) from *n* sequenced genomes (Fig. 3). The core genes shared within the two groups (discussed in detail below) levelled off quickly as new genomes were added to the analysis, suggesting that this small sample size of diverse T4-like phages is adequate for determining the core. As expected, the total number of unique genes identified (the pan-genome) steadily increased with the number of available genomes in both cases. The size of the pan-genome reached 1388 and 1445 genes for the cyanophages and non-cyanophages respectively (Fig. 3A and B). The rate of increase of both pan-genomes as more genomes are added to the analysis is far from saturated, indicating the existence of a much larger and diverse gene pool than has been captured by the 26 sequenced genomes. Interestingly, however, the cyanophage pan-genome showed a slower rate of increase (Fig. 3A) than that of the non-cyanophages (Fig. 3B).

#### The T4 core, shared by all 26 T4-like phage genomes

Thirty-eight genes were common to all 26 genomes (Fig. 2A, Table S2), while also maintaining remarkable synteny (Fig. S2). The only exceptions to the synteny included a large inversion among the cyanophages relative to the non-cyanophages, and a few notable smaller-scale breaks in synteny likely due to mobile element activity (see *Genome evolution in the cyanophages*). Of the 38 genes shared by all the genomes, 27 form sequence-based orthologous groups (T4-GCs; see *Experimental procedures*), while the remaining 11 display enough sequence divergence that these functional homologues are placed into multiple T4-GCs. While the number of core genes decreased as more T4-like phage genomes were added to these analyses (Miller *et al*., 2003b; Mann *et al*., 2005; Sullivan *et al*., 2005; Comeau *et al*., 2007; Weigele *et al*., 2007; Millard *et al*., 2009), it appears that we have now adequately defined the core (Fig. 3) and that these *T4 core* functions involve appropriating host metabolic machinery, replicating the viral genome during infection, and building the viral particles.

#### Nearly ‘T4 core’ genes

Beyond the *T4 core* genes are a handful of noteworthy *nearly core* genes, i.e. those present in at least 22 genomes across the 26 T4-like phage genomes. An analysis of the patterns of their distributions makes these genes potentially useful targets for experimental functional identification, or indicators of novel functions in particular groups of isolates. This set of genes includes the gp51 baseplate hub assembly catalyst (missing only in Aeh1, but note that cyanophage gp51 are only ∼20% of the length of non-cyanophage gp51, Fig. S3), nucleotide metabolism and recombination/repair genes *uvsX*, *uvsY* (both missing in the same three phages – 44RR, PHG25, PHG31), and the gp59 loader of gp41 helicase (found in 22 of 26 T4-like phages).

#### The non-cyanophage core, shared by all 10 non-cyanophage genomes

In addition to the 38 genesshared by all the genomes, the non-cyanophage genomes shared an additional 32 *non-cyanophage core* genes (Fig. 2A, Table S3), giving this group a shared core of 70 genes down from the most recent estimate of 90 core genes shared among three non-cyanophage T4-like genomes (Comeau *et al*., 2007). All but six of the 32 *non-cyanophage core* genes have been functionally annotated in coliphage T4 (Miller *et al*., 2003a), and the larger proteins such as structural proteins gp7, gp10 and gp12 were so divergent as to be comprised of up to nine T4-GC clusters (Table S3). Many of these additional *non-cyanophage core* genes encode functions involved in ‘host specialized’ viral structure (e.g. tail fibres) and DNA replication machinery. We expect that experiments targeting functional annotation of shared hypothetical proteins in the cyanophages will reveal that many of these host specific functions exist in the cyanophages, but as divergent gene copies. In contrast, other genes, such as *nrdD* and *nrdH* genes, are likely only relevant to the specific habitat of some of these non-cyanophages (e.g. anaerobic sewage).

#### The cyanophage core, shared by all 16 cyanophage genomes

Twenty-five genes were shared by all 16 cyanophages (Fig. 2A, Table S4), in addition to the 38 that form the *T4 core*, for a total of 63 genes shared across the cyanophages which now appears to be a stable shared gene set among the T4-like cyanophages (Fig. 3A). All but one of these 25 *cyanophage core* genes was absent from the non-cyanophages (Table S4). This exception is the *phoH* gene that was found in only one of the other genomes – the marine vibriophage KVP40 – and may represent an adaptation valuable both for infection of cyanobacteria, but also more generally of marine hosts (e.g. marine vibrios) rather than a cyanophage-specific function. However, some do appear cyanophage-specific, such as the previously described cobalamin biosynthesis protein (*cobS*), or photosynthesis proteins for the central photosystem II reaction centre protein (*psbA*) and highlight-inducible proteins (*hli*) (Mann *et al*., 2005; Sullivan *et al*., 2005; Weigele *et al*., 2007). Other *cyanophage core* genes include proteins that likely encode basic phage functions, such as a heat shock family protein (*hsp20*) that might be important for scaffolding during maturation of the capsid, and two experimentally determined virion structural proteins (T4-GCs 15, 190). In addition, the *cyanophage core* includes phosphate stress-induced protein (*phoH*), pyrophosphatase (*mazG*), and dioxygenase proteins (T4-GCs 101, 155 with similarity to PFAM PF05721) that are discussed in greater detail below. The remaining genes encode hypothetical proteins of unknown function. An understanding of the functions of these proteins, combined with a deeper understanding of the PhoH and MazG proteins (discussed below) should further elucidate the nature of cyanophage–host interactions.

#### Notable cyanophage core and nearly cyanophage core genes

The *cyanophage core* gene *mazG* has received a lot of recent attention. In *E. coli*, MazG appears to be a regulator of nutrient stress and programmed cell death (Magnusson *et al*., 2005; Gross *et al*., 2006; Lee *et al*., 2008), as its dNTP pyrophosphatase activity acts on the signalling nucleotide guanosine tetraphosphate (ppGpp) to regulate up to one-third of *E. coli* genome (Traxler *et al*., 2008). In cyanophages, MazG is also thought to act as a global transcriptional regulator through modulation of ppGpp levels, which may extend the period of cell survival under the stress of phage infection (Clokie and Mann, 2006; Weigele *et al*., 2007). However, MazG enzymes are highly specific for non-canonical NTPs, suggesting that identifying their substrates likely requires solving crystal structures along with activity and binding assays for each new enzyme (Galperin *et al*., 2006). Thus the cyanophage MazG substrate should be cautiously interpreted.

Regardless of function, the *mazG* gene has a notable distribution among T4-like cyanophages. Recently, it was found by PCR screens to be present in nine out of 17 cyanophage myovirus isolates (Bryan *et al*., 2008). In contrast, all 16 of our cyanophage myovirus genomes contained this gene. While this difference could be real, it likely reflects the limitations of PCR screening, which can only reveal the presence of a gene whose sequence is known (Millard *et al*., 2004; 2009;). Consistent with this interpretation, Bryan and colleagues (2008) observed > 99% identity among their sequenced *mazG* PCR products obtained from geographically diverse isolates, while the *mazG* sequences of our genomes showed marked sequence divergence (Fig. S4). Nonetheless, in agreement with Bryan and colleagues (2008), our analyses also suggest that *mazG* arose from outside the cyanobacteria (Fig. S4), as opposed to most other ‘host’ genes in cyanophages which originate from their host strains (Sullivan *et al*., 2005; Williamson *et al*., 2008), and is most closely related to the genes from *Choloroflexus*.

Finally, in addition to the core *mazG* gene, nine genes are *nearly cyanophage core* genes as they are found in 15 of the 16 cyanophages, missing only in the anomalous S-PM2 phage (see below).

#### Genome variability of two co-isolated cyanophages

**Fig. 4:**
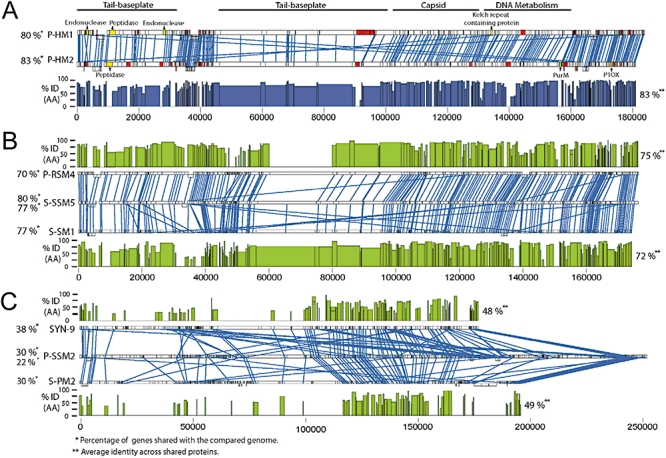
Whole-genome pairwise comparisons across the bounds of the cyano T4 phage genome diversity are examined here. In all three panels, two genomes are compared where lines between the genomes connect homologues, coloured ORFs indicate genes that are unique to one genome or the other, and the per cent identity of each ORF is plotted in the lower half of each panel. Pairwise genome comparisons are presented for (A) two co-isolated cyanophages, P-HM1 and P-HM2, as well as (B) the three closest non-co-isolated phages, P-RSM4, S-SSM5 and S-SM1, and (C) the three most distant non-co-isolated phages, P-SSM2, S-PM2, Syn9, among the 16 sequenced cyanophage genomes.

To explore genomic diversity among spatially coexisting phages capable of infecting the same host, we included in this sequencing project two phages isolated from the same water sample on the same host strain (Fig. 4A). These two cyanophages, P-HM1 and P-HM2, are highly syntenic and share 200 of 241 and 242 genes, respectively, whose protein sequences are on average 83% identical (Fig. 4A). In contrast, pairwise genome comparisons showed that among the non-co-isolated cyanophages, the genomes share as much as 77–80% of their genes with average identity 72–75% (Fig. 4B) or at the least 22–33% of their genes, with only 48–49% average identity (Fig. 4C).

Further comparison of the two co-isolated phage genomes (Fig. 4A) showed that, while the protein identity of orthologues shared between P-HM1 and P-HM2 averaged 83%, there was an enormous range (21–100%) in this value. On the one hand, orthologue identities could be quite low (21–32%) and include hypothetical proteins and even proteins that are part of the *cyanophage core* such as CoA-dioxygenase and Hsp20. On the other hand, orthologue identities could be quite high (100%) for other *cyanophage core* proteins such as Hli03, gp55, as well as for non-conserved hypothetical proteins such as T4-GCs 429, 542 and 559, which are found only in a subset of *Prochlorococcus* phages. The non-shared proteins, predominately hypotheticals, were notably clustered into distinct regions of the genomes (Fig. 4A) akin to cyanobacterial genomic ‘islands’ (*sensu*Coleman *et al*., 2006). In addition to hypotheticals, the non-shared gene set did include some annotation (Table S5): a purine biosynthesis gene (*purM*) and plastoquinol terminal oxidase (PTOX, described further below) are unique to P-HM1, while a pair of endonucleases and a Kelch-repeat-containing protein are unique to P-HM2. In addition, peptidase genes were present in syntenic genomic locations in both phages (Fig. 4A) even though their sequences had diverged to the point of forming separate gene clusters (T4-GCs 573, 452). These phages also contain 70 genes found in both phages, but not in any of the other sequenced cyanophages. These 70 genes encode an S8 peptidase (T4-GC518), glycine dehyrogenase (T4-GC540), two asparaginyl beta-hydroxylases (T4-GCs 536, 546), an acyl carrier protein (ACP, T4-GC457) and its synthetase (ACPS, T4-GC500), a terminal quinol oxidase (T4-GC555), taurine catabolism dioxygenase (T4-GC447), and hypotheticals. That genes encoding these proteins were found only in these two co-isolated MED4-infecting phages might provide clues to requirements for infection of *Prochlorococcus* MED4 in these Hawaii Ocean waters.

#### The cyanophage-exclusive, but not universal, gene set

We identified 143 genes that occurred in four or more of the 16 cyanophage genomes, but were absent from all of the non-cyanophage genomes (summarized in Table 2). Ninety-six of these encode hypothetical proteins, but others encode a diversity of photosynthesis (*psbD*, *petE*, *petF*, PTOX, *pebS*), phosphate stress (*pstS*), carbon metabolism (*talC*, CP12) and virion structural (24 genes) proteins, the functions of which are consistent with our notion of a cyanophage lifestyle. Some of these are discussed further below.

**Table 2:** Summary of the 143 ‘non-core’ genes that are enriched in cyanophages (found in > 3 genomes), but are absent from non-cyanophages

Gene present in # genomes	# of genes	Prominent functions (remainder are hypothetical proteins)
4	26	*petF*, *ho1*, carbamoyltransferase, *pebS*, 5 virion structural proteins
5	17	Enase VII, HN, DUF120
6	14	*prnA*, *speD*, carboxylesterase, 3 virion structural proteins
7	12	2 virion structural proteins
8	17	*purM*, 3 virion structural proteins
9	15	*pstS*, PTOX, 6 virion structural proteins
10	5	*petE*, 1 virion structural protein
11	5	all hypothetical proteins
12	11	*psbD*, *cpeT*, 1 virion structural protein
13	3	*denV*
14	6	N6A-methylase, helicase, 2OG-FeII oxygenase, 1 virion structural protein
15	12	*talC*, CP12, DUF680, endonuclease, 1 virion structural protein

#### The *Synechococcus*-enriched gene set

We found no genes that were universal and exclusive to the 10 cyanophages isolated on *Synechococcus*. However, there were 48 genes that occurred in three or more of this phage set, and occurred in no others (Table S6). Notably, these genes clustered in four ‘hot-spot’ regions of the genomes: (i) near gp5 with tRNAs, (ii) with small genes between gp46 and gp25, (iii) between gp16 and gp17 (previously identified by Millard *et al*., 2009) and (iv) near *psbA*, again commonly with numerous tRNAs (Fig. 1). Although 42 of these 48 genes encode hypothetical proteins, two are involved in carbon metabolism (*zwf*, *gnd*– discussed below), three had PFAM domains that suggested function (PA14 carbohydrate-binding domain, DUF1583, and SAICAR synthetase purine biosynthesis), and one is a virion structural protein (T4-GC969; see *Experimentally identified cyanophage structural proteins*).

#### The *Prochlocococcus* T4 core and enriched gene set

Two genes were universal and exclusive to cyanophages isolated on *Prochlorococcus* (Table S7). These *Prochlorococcus* T4 core genes encode a possible photosystem II PsbN (Pfam domain PF02468, T4-GC163, no functional role has yet been determined for PsbN), and a hypothetical (T4-GC285). As well, there were 16 genes that occurred in three or more of this phage set, and occurred in no others (Table S7). These clustered in ‘hot-spot’ genome regions homologous to those described above for the *Synechococcus*-enriched genes (Fig. 1), and include genes encoding a highlight-inducible protein (T4-GC436), a phycocyanobilin biosynthesis protein (*pcyA*, T4-GC413) and 14 hypothetical proteins. Finally, two hypothetical proteins were universal among the six *Prochlorococcus* phages, but not exclusive to them (T4-GC082 also found in S-SSM7 and S-SSM5; T4-GC224 also found in S-SSM7).

#### The odd cyanophage out

*Synechococcus* cyanophage S-PM2 appears quite distinct from the 15 other cyanophages. First, its %G+C content is similar to that of a *Prochlorococcus* phage (Table 1). Second, S-PM2 lacks nine *nearly cyanophage core* genes that are found in all of the 15 other cyanophages, and two genes found in 14 of the 15 other cyanophages. In contrast, only one other cyanophage (P-SSM2) is missing even a single gene (T4-GC424) that is ubiquitous among the other 15 cyanophages. Among the genes ‘missing’ in S-PM2 are eight hypothetical genes, an endonuclease and two carbon metabolic proteins (transaldolase and CP12 = T4-GCs 63, 337). Finally, S-PM2 contains only seven of the 45 ‘*Synechococcus*-enriched’ phage genes, whereas, other than *Synechococcus* phage S-SSM7 (containing only two), the rest of the cyanophage genomes contained 18–27 (average = 23) of the 45 *Synechococcus* phage-enriched genes. Given the data set at hand, we cannot identify any variables that might explain why this particular phage is so different from the others.

### Sporadically distributed ‘host’ genes – a link to cyanobacterial phage – host ecology and evolution

In contrast to the syntenic, widely distributed sets of genes described above, a number of genes exhibit more sporadic distributions across the cyanophage genomes (Table 3), and these are likely driving niche differentiation of cyanophage–host systems (Lindell *et al*., 2004; Coleman *et al*., 2006). Here we highlight a few of these genes, the putative functions of which can be readily connected to known variables in cyanobacterial and cyanophage ecology.

**Table 3:** Summary of cyanobacterial specific sporadically distributed genes among 16 T4-like cyanophages

T4-GC#	Functional annotation	P-SSM2	P-SSM4	P-HM1	P-HM2	P-RSM4	P-SSM7	S-PM2	Syn9	Syn19	Syn33	Syn1	S-ShM2	S-SM2	S-SSM7	S-SSM5	S-SM1
*Photosynthesis*																	
440	PsbD = photosystem II D2 protein	–	1077	1077	1077	–	–	1062	1056	1056	1056	1056	1056	1056	–	1056	1056
270 + 271 + 274 + 436	Hli = highlight-inducible proteins	114|144|105	135	135	201	219	114	–	–	–	–	–	–	–	–	–	–
404	PTOX = plastoquinol terminal oxidase	–	501	504	–	504	–	–	507	504	–	–	–	504	504	504	504
225	PetE = plastocyanin	345	–	–	–	–	–	–	324	339	324	324	324	324	369	324	351
276	PetF = ferredoxin	294	–	–	–	–	288	–	–	–	291	–	–	294	–	–	–
411	SpeD = *S*-adenosylmethionine decarboxylase	–	306	–	–	–	–	333	–	–	–	327	–	336	–	342	336
338	CpeT-like protein	–	444	405	405	456	432	528	486	462	459	–	447	–	–	459	453
55	PebS = phycoerythrobilin biosynthesis	702	–	624	624	–	–	–	–	–	–	–	–	–	648	–	–
413	PcyA = phycobilin biosynthesis	–	690	–	–	729	717	–	–	–	–	–	–	–	–	–	–
286 + 1398	Ho1 = Haem oxygenase	702	–	693	693	–	–	–	–	–	–	–	–	–	582|165	–	–
615	Hyp. with ferrochetalase domain	–	–	–	–	591	573	609	543	–	–	–	546	732	–	591	531|600
104 + 240 + 412 + 611	2OG-Fe(II) oxygenase superfamily	594|576	567|591|567	–	–	573|711|546|558|600	648|576|579|624	582|717|603	570|606|552	564|597|573	594|594|579	729|609|648	591|600	621	696|618	567|537|558|546|768	582|612|621|573|591
*Carbon metabolism*																	
920	Gnd = 6-phosphogluconate dehydrogenase	–	–	–	–	–	–	–	1038	1038	–	1038	1023	1038	–	1041	1041
921 + 1021	Zwf = glucose-6-phosphate dehydrogenase	–	–	–	–	–	–	–	1446	1440	276	303	306	1440	–	1443	1437
337 + 63	CP12 = carbon metabolic regulator	267	228	213	213	249	246	–	213	285	228	231	228	228	228	228	228
239	TalC = transaldolase	648	654	675	675	678	687	–	702	660	660	648	663	648	654	681	747
*Phosphate stress*																	
1254	PhoA = alkaline phosphatase	–	–	–	–	–	–	–	–	–	–	–	–	1263	–	–	1356
	PstS = ABC-type phosphate transport system																
243	Substrate binding protein	966	966	–	–	987	966	–	–	981	–	–	–	963	978	981	990
*Other functions*																	
212	PrnA = tryptophan halogenase	1458	–	–	–	–	–	–	1593|1524	–	–	1437	1116	–	1104|1488	1167	–
425	S-layer domain protein	–	573	–	–	573	633	–	–	–	–	–	–	–	564	573	573
438	Carboxylesterase	–	414	375	429	135	–	–	–	–	–	–	–	276	399	–	–
395	HN = haemaglutanin neuraminidase	–	474	474	492	474	–	–	–	–	–	–	–	–	489	–	–
303 + 721 + 1350	Carbamoyltransferase	1803	–	–	–	1665	1524	–	–	–	–	–	–	–	1803|1536|1575	1665	–
194	tRNA ligase	741	–	–	–	–	798	–	–	–	–	–	–	–	–	–	–

Presence of the gene occurring in a particular genome is indicated by its size being listed (bp) rather than the lack of the gene indicated by ‘–’. A ‘|’ separates multiple copies of a gene that occur in the same genome.

#### Phosphorus utilization genes

**Fig. 5:**
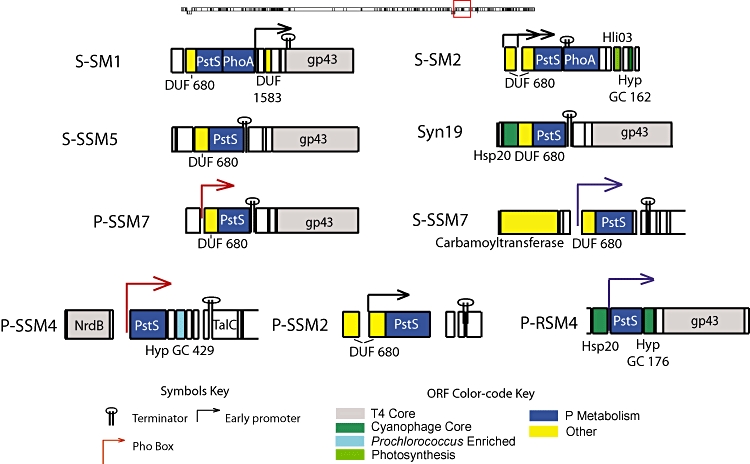
Close-up genome representation of the phosphate genes cluster from cyanophages. Genomic features are as described in Fig. 1. To orient the reader to the genome location of the cluster being portrayed, a box is drawn in a reference genome for each or a group of similarly placed phage gene clusters.

Phosphorus often limits productivity in oligotrophic marine systems, and cyanophages have been shown to contain the phosphate stress gene, *pstS* (Sullivan *et al*., 2005), which shuttles phosphate from the outer to the inner membrane in cyanobacteria. Two *Prochlorococcus* T4-like phages isolated from the Sargasso Sea have been shown to encode the gene, while it was not found in two *Synechococcus* T4-like phages from coastal waters (Mann *et al*., 2005; Sullivan *et al*., 2005; Weigele *et al*., 2007, but also see *Note added in proof*). This raises the question of whether *pstS* distribution is driven by host strain, source waters, or both. Here we observed that homologues of the *pstS* gene were found in nine of the 16 cyanophages (Table 3). While the nine phages were isolated on six different *Prochlorococcus* and *Synechococcus* host strains, all originated from low-nutrient waters, where phosphorus is likely in short supply. Thus it appears that the source waters used for phage isolation are more important than host strain for predicting the presence or absence of *pstS* in the phage genome – a relationship that has been observed in metagenomic analyses of surface ocean samples (Williamson *et al*., 2008). In addition to the gene itself, we also identified transcriptional regulatory machinery flanking all nine *pstS* genes, including promoters (Fig. 5, Fig. S5) and terminators (Fig. 5). No single regulatory solution was apparent across the genomes. Interestingly, two of the phages (S-SM1, S-SM2) contained *phoA*, which encodes an alkaline phosphatase, next to *pstS* (Fig. 5). If functional, this could facilitate access to organic phosphorus.

Homologues of *phoH*, a gene that belongs to the phosphate regulon in *E. coli* and encodes a putative ATPase, were found in all 16 cyanophages as well as the marine T4-like vibriophage KVP40 (Miller *et al*., 2003b). This gene is absent from some other non-T4-like marine cyanophages [e.g. podoviruses P-SSP7 (Sullivan *et al*., 2005) and P60 (Chen *et al*., 2002), siphovirus P-SS2 (Sullivan *et al*., 2009)], but present in other marine phages, i.e. the distant T7-like roseophage SIO1 (Rohwer *et al*., 2000); thus clear patterns are not evident. We had previously described (Sullivan *et al*., 2005) such phage-encoded *phoH* genes as apparent parts of a multi-gene family with divergent functions from phospholipid metabolism and RNA modification (COG1702 *phoH* genes) to fatty acid beta-oxidation (COG1875 *phoH* genes) (Kazakov *et al*., 2003); indeed the function of the *phoH* gene, particularly in cyanobacteria, remains unclear. For example, under phosphate stress, the gene has been shown to be upregulated in *E. coli* (Wanner, 1996) and *Corynebacterium glutamicum* (Ishige *et al*., 2003), downregulated in *Synechococcus* WH8102 (Tetu *et al*., 2009), and unaffected in at least two *Prochlorococcus* strains (Martiny *et al*., 2006). The uniform presence of the gene in the T4-like cyanophages, combined with this mosaic of other patterns of distribution and expression, is intriguing.

#### Carbon metabolism genes

The distribution of carbon metabolism genes among the cyanophage genomes (Table 3) suggests that many have co-opted critical enzymes to access reducing power from glucose via the pentose phosphate pathway (PPP). All but S-PM2 (Mann *et al*., 2005) have the transaldolase gene (*talC*), thought to be important in mobilizing stored carbon through the PPP, and observed previously in three T4-like cyanophage genomes (Sullivan *et al*., 2005; Weigele *et al*., 2007). These phages also carry the gene that encodes CP12, a cyanobacterial regulatory protein that inhibits several Calvin cycle enzymes, promoting carbon flux through the PPP at night (Tamoi *et al*., 2005). We recently identified a homologue of CP12 in *Prochlorococcus*, whose identity was strengthened by a diel expression pattern consistent with this function (Zinser *et al*., 2009). This led to the identification and analysis of *cp12* in these phage genomes, with the diel expression patterns of PPP genes (Zinser *et al*., 2009) informing their possible role in cyanophages (L.R. Thompson *et al*., in preparation). In addition to carrying *talC* and *cp12*, eight *Synechococcus* cyanophages encode two other pentose phosphate pathway enzymes, of varying sequence conservation (see below), which generate NADPH: *zwf*, a glucose-6-phosphate dehydrogenase, and *gnd*, a 6-phosphogluconate dehydrogenase. The existence of as many as four PPP genes in some phages suggests that this pathway is critical to cyanophage infection. We suggest that this may be due either to increased reducing power stored in carbon substrates or to the production of ribulose-5-phosphate which may alleviate bottlenecks in nucleotide metabolism.

#### Nitrogen metabolism genes

A well-known cyanobacterial response to nitrogen stress is the degradation of phycobilisomes through the activity of the non-bleaching protein NblA. While the *nblA* gene has been observed in a freshwater cyanophages (Yoshida *et al*., 2008), this gene has not been found in marine cyanobacteria and has not been observed among marine cyanophage. Here we propose cyanophage involvement in host nitrogen metabolism that likely involves a response to intracellular levels of 2-oxoglutarate (2OG) in the host. Ammonium, the preferred nitrogen source for cyanobacteria, is assimilated through incorporation into a 2OG carbon skeleton. Ammonia limitation thus results in 2OG accumulation in the cell, which serves as an indicator of nitrogen status (Irmler *et al*., 1997; Forchhammer, 1999; Muro-Pastor *et al*., 2001). DNA binding of the global nitrogen regulator, NtcA, is 2OG-dependent such that NtcA is inactive when 2OG is limiting and the cell has excess available nitrogen, whereas the opposite is true under nitrogen stress conditions (Schwartz and Forchhammer, 2005).

**Fig. 6:**
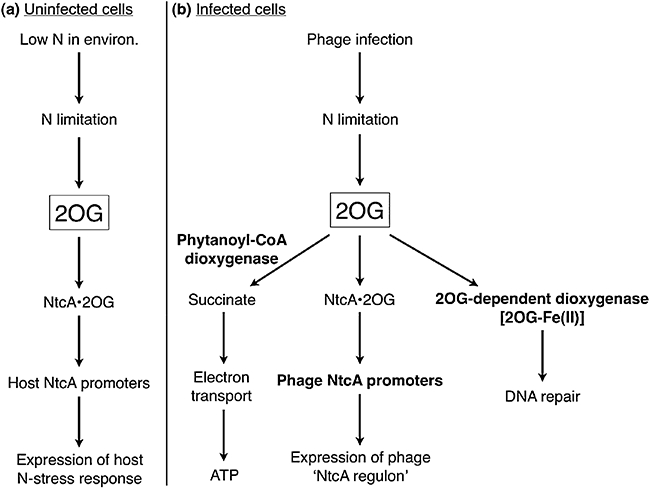
Proposed role of 2-oxoglutarate (2OG) during cyanophage infection. A. In uninfected cyanobacteria, nitrogen limitation causes 2OG to accumulate, leading to 2OG-dependent binding of NtcA to promoters of nitrogen-stress genes, resulting in their expression. B. Phage infection draws down cellular nitrogen causing N-stress and likely leading to 2OG accumulation. Several cyanophage-encoded enzymes (in bold) suggest that increased 2OG may facilitate phage infection. First, a putative phytanoyl-CoA dioxygenase may convert 2OG to succinate, a major electron donor to respiratory electron transport in cyanobacteria (Cooley and Vermaas, 2001) thus potentially generating energy for the infection process. Second, 2OG-dependent dioxygenase [2OG-Fe(II)] superfamily proteins may function in cyanophage DNA repair (Weigele *et al*., 2007). Third, cyanophage genomes have multiple NtcA promoters driving genes encoding diverse functions – possibly exploiting the host NtcA-driven N-stress response system.

Three features of the cyanophage genomes suggest that they modulate 2OG levels to stimulate NtcA activity as needed to promote phage gene expression (Fig. 6). First, all 16 genomes contain numerous NtcA binding sites (1–16 per genome; average = 8.9), which apparently promote a diversity of both T4 phage and cyanophage functions (Fig. 1). Second, 14 of the 16 genomes contain numerous 2OG-FeII oxygenase superfamily proteins (Table 3). Third, all 16 cyanophages contain at least one and often numerous hypothetical proteins with possible phytanoyl-CoA-dioxygenase domains, (Table S4), which may act on 2OG, in this case as oxidoreductases.

#### Photosynthesis-related genes

Cyanophage-encoded phycobilin biosynthesis genes have previously been shown to be expressed during infection (*pebS*) and functional *in vitro* (*pcyA*, *pebS*, *ho1*; Dammeyer *et al*., 2008). These genes, *pcyA*, *pebS*, *ho1*, occur in three, four and four of the 16 cyanophage genomes respectively (Table 3). As well, the *cpeT* gene previously observed in S-PM2, S-RSM4 and Syn9 (Mann *et al*., 2005; Weigele *et al*., 2007; Millard *et al*., 2009) is found in 12 of the 16 cyanophage genomes examined here (Table 3). Notably, the *cpeT* gene in marine cyanobacteria is part of a phycoerythrin *cpeESTR* operon, so the role of the cyanophage-encoded copy remains unresolved given the lack of *cpeESR*.

**Fig. 7:**
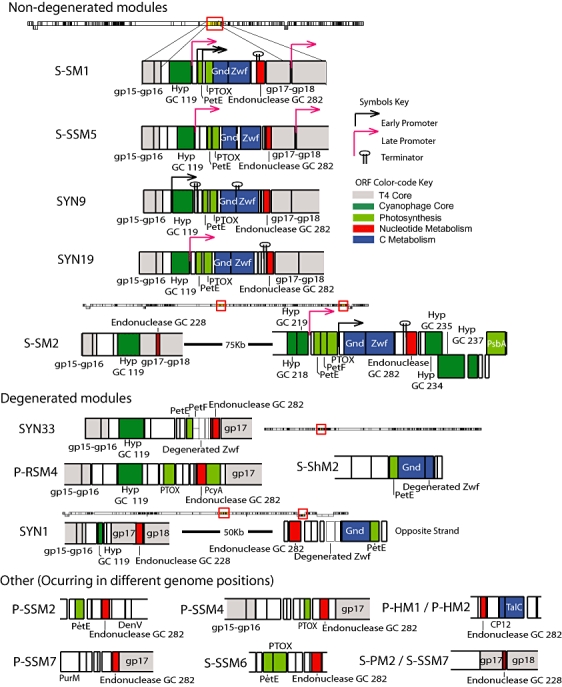
Close-up genome representation of the carbon metabolic gene cluster from cyanophage genomes. Genomic features are as described in Fig. 1, and genome location orientation is as described for Fig. 5.

Sporadically distributed among the cyanophage genomes are two electron transport genes, *petE* and PTOX, which encode proteins that commonly co-occur with the carbon metabolism genes (*zwf* and *gnd*, described above) as part of a hypothesized mobile gene cassette (Fig. 7) and likely prevent electrons from backing up and damaging photosynthetic reaction centres. The *petE* gene encodes plastocyanin, and has previously been described in cyanophages (Sullivan *et al*., 2005; Weigele *et al*., 2007; Millard *et al*., 2009). PTOX proteins are normally associated with carotenoid desaturation (Kuntz, 2004), but in cyanophages are hypothesized to help maintain balanced pools of ATP and NADPH in infected host cells (Weigele *et al*., 2007; Millard *et al*., 2009). Consistent with this hypothesis, a marine *Synechococcus* was shown recently to use PTOX-related oxidases to shunt off excess inter-photosystem electrons to oxygen rather than to PSI (Bailey *et al*., 2008), which would significantly impact ATP/NADPH pools. This alternate electron flow was thought to be particularly important under Fe-limiting conditions when PSI/PSII reaction centre ratios drop (Bailey *et al*., 2008). Consistent with this observation, PTOX genes are abundant in open ocean surface water microbial metagenomes (McDonald and Vanlerberghe, 2005), and are found in many surface water oligotrophic *Prochlorococcus* (AS9601, MIT9301, MIT9215, MIT9312, MED4, NATL1A, NATL2A) and *Synechococcus* (BL101, WH8102, CC9902) isolates (data not shown), although lacking in their less Fe-limited counterparts from deeper or coastal waters (e.g. LL *Prochlorococcus* and *SynCC9605*).

### Experimentally identified cyanophage structural proteins

To maximize our ability to annotate cyanophage structural proteins, we analysed the proteome of S-SM1 experimentally, and detected multiple peptides from 41 proteins in the purified S-SM1 virion (Table S8, which includes the *Synechococcus*-enriched gene T4-GC969 described above). These 41 proteins in S-SM1 and their orthologues in the other 15 cyanophage genomes are designated on Fig. 1 as ORF ‘underlining’, along with the data from two other T4-like phage proteomics projects [S-PM2 (Clokie *et al*., 2008) and Syn9 (Weigele *et al*., 2007)]. Notably, these include nine proteins known to be encoded in the S-PM2 genome, but not detected in the virion (Clokie *et al*., 2008). These nine newly detected proteins encode homologues of seven coliphage T4 structural proteins (gp 4, 5, 14, 21, 25, 48, 53), as well as two cyanophage core proteins, including a putative citidylyltransferase (T4-GC190) and a hypothetical protein (T4-GC15). We also identified 18 hypothetical proteins which expand the existing data set of T4-like structural proteins; all of them need structural/functional assignments. We note that 10 virion structural proteins have similar distributions among nine of the cyanophage genomes (Table S8); perhaps these proteins are functionally linked, T4 phage structural components.

### Genome evolution in the cyanophages

As discussed above, the ‘cyanophage core’ genes are remarkably syntenic across the 16 cyanophage genomes (Fig. S2), suggesting that most of these cyanophage specialization genes are vertically transmitted and part of general T4 phage strategies for infection of ocean cyanobacteria. Twenty-four ‘core’ genes among non-cyanophages were previously inferred to be vertically transmitted and resistant to horizontal gene transfer (Filee *et al*., 2006; Comeau *et al*., 2007). It is thought that such genes might be resistant to horizontal gene transfer due to complexity of the T4 protein–protein interactions required for the complex structure (Leiman *et al*., 2003) and metabolic function (Miller *et al*., 2003a) of phage T4 and by analogy, the T4-like phages. In contrast, phylogenies of non-core genes in the T4-like non-cyanophages have conflicting topologies which are interpreted to be due to horizontal gene transfer (Filee *et al*., 2006). Similarly, our cyanophage core genes are remarkably syntenic, presumably also due to vertical transmission from phage to progeny phage, and the few exceptions to this synteny may be due to the activity of mobile genetic elements (Fig. S2). Such mobile element activity in T4 phages has been previously observed in coliphage T4 (Miller *et al*., 2003a), as well as ocean cyanophages ranging from T4-like phages (Zeng *et al*., 2009) to siphoviruses (Sullivan *et al*., 2009). Specifically, tRNA genes co-occur with many of these altered non-syntenic regions of the genome (Fig. 1), and may serve as substrates for site-specific recombination by mobile genetic elements (Williams, 2002; Campbell, 2003).

The carbon metabolism genes carried by cyanophages may be particularly influenced by the movement of mobile gene cassettes. For example, *zwf* and *gnd* co-occur in the genomes of eight phages isolated on *Synechococcus* as part of a possible mobile gene cassette (Fig. 7): five contain paired, full-length, apparently functional gene cassettes in varied genome locations, while three contain variously degraded gene cassettes including remnants of *zwf* genes (Fig. S6). The other genes in the cassette include two photosynthetic electron transport genes (*petE* and PTOX, see above), a hypothetical protein (T4-GC119) and an endonuclease, which may at some point have mobilized the cassette as described below. Notably, a ninth genome (*Prochlorococcus* phage P-RSM4) lacks *zwf* and *gnd* entirely, but appears to have remnants of the rest of this cassette (Fig. 7).

The endonucleases in this region are notable as, in phage T4, such genes are known to be part of selfish DNA elements known as intronless homing endonucleases in both coliphages (Belle *et al*., 2002; Liu *et al*., 2003) and T4 cyanophages (Zeng *et al*., 2009). It is plausible that such selfish genes might lead to highly recombinogenic regions in the T4 genome as the nuclease errs and yields double-strand breaks. Here we observe two forms of endonucleases (Fig. S7) – one of which contains sequences with distant homology to this confirmed homing endonuclease (T4-GC228) where only one member (from P-SSM2) contains the catalytic residues identified by Zeng and colleagues (2009); the second contains sequences that lack any homology to the experimentally determined cyanophage T4 homing endonuclease (T4-GC282). Notably, this endonuclease-flanked cassette is located in variable locations in the genomes (Fig. 7). In four of the genomes the cassette appears in the same gp17–gp18 region that Millard and colleagues (2009) recently described as a hypervariable region. In a fifth genome, S-SM2, the cassette appears near *psbA*, where it is interrupted by a second gene cassette (the hypothetical T4-GCs cluster described below). The four additional genomes contain degraded forms of this cassette in varied genome locations. Beyond this carbon metabolism cassette, we note that additional carbon metabolism genes, *talC* and *cp12*, occupy variable genome positions ranging from locations in the 5′- or 3′-end of the *psbA* region or near gp5, but are often proximal to tRNAs (Fig. S8).

Two other classes of gene cassettes carry signatures of mobility in these genomes. First, a cluster of five hypothetical proteins (T4-GCs 218, 219, 234, 235, 237), often associated with a plasmid stability protein, was found in all but one (S-PM2) of the cyanophages (Fig. S9). This cluster was similarly positioned and structured across nine genomes, but varies across the other six genomes. We hypothesize that these proteins are clustered for functional reasons, and that the plasmid stability protein may offer mobility of the gene cassette. Second, large clusters of lipopolysaccharide (LPS) genes are present in the larger cyanophage genomes (Fig. 1) located either near *hli03* (S-SSM7, S-SM2, P-SSM2) and/or near *phoH* (P-SSM2), again proximal to tRNAs. It is not known whether these LPS biosynthesis genes are functional or are simply ‘stuffer DNA’ for headful packaging in these larger genome phages. However, seven LPS genes co-occur in three phages that were isolated 2 years apart using source waters hundreds of miles distant from each other (T4-GCs 260, 265, 266, 304, 305, 307, 308 all occur in P-SSM2, S-SM2, and S-SSM7). Either a recent transfer event occurred across these three disparate phages, or, perhaps more likely, these LPS genes are functionally linked and represent convergent evolution.

## Conclusion

With this expanded data set we have been able to better define the T4-like phage core genome. The challenge now is to examine more closely the non-core genes required for infection of different hosts and environments. Our analysis reinforces the importance, for cyanophage, of carrying genes involved in the light reactions of photosynthesis, the pentose phosphate pathway, and phosphorus acquisition. In addition, we reveal a possible link to host nitrogen metabolism. Finally, the genome-wide comparison of two phages isolated on the same host from the same sample, offers a first look at *intra*-population genomic variability that is a critical first step to understanding the biogeography of phage diversity.

## Experimental procedures

### Phage isolation, purification, DNA extraction and sequencing

Twelve cyanophages were isolated (Waterbury and Valois, 1993; Sullivan *et al*., 2003; Sullivan *et al*., 2008), then concentrated and purified for genomic DNA extraction either by CsCl purification (details in Lindell *et al*., 2004) or using a Lambda Wizard DNA kit (Promega Corp., Madison, WI) directly on phage lysates. This kit precipitates phage particles using a polyethylene glycol solution, followed by DNA extraction using a diatomaceous earth-based resin (Promega Corp., Madison, WI). Total DNA yields were consistently higher using the Wizard DNA kit than using CsCl-purified particles (1–2 µg from 250 ml of lysate versus nanograms from 2 l of lysate). Although host DNA contamination was significant (ranged 11.4–77.5% of total reads) in the Wizard DNA kit preps due to the less rigorous purification, host reads could be filtered out during phage genome assembly. These methods are described in detail elsewhere (Henn *et al.*, 2010).

### Construction and pyrosequencing libraries

Pyrosequencing libraries preparations are described in Henn and colleagues (2010). Briefly, 100 µl of cyanophage genomic DNA (1 ng to 2.2 µg) was sheared using Covaris AFA technology and the following conditions: time = 240 s, duty cycle = 5, intensity = 5; cycles per burst = 200 and temperature = 3°C. Post-shearing, the DNA was concentrated and fragments less than 200 bp were removed using AMPure PCR purification beads (Agencourt Bioscience Corporation, Beverly, MA). The DNA shearing profile was determined by running 1 µl of the samples on the Agilent Bioanalyser 2100 using a DNA 1000 chip (Agilent Technologies, Santa Clara, CA) with the optimal size for library construction being 1.2–1.5 kb fragments. The sheared DNA was then used for pyrosequencing library construction with reagents provided in the GS 20 Library Preparation Kit (454 Life Sciences, Branford, CT) according to manufacturer's instructions for fragment end-polishing, adaptor ligation and library immobilization reactions but slightly modified for the clean-up steps, which were performed with the addition of 1.8× AMPure beads.

### Genome assembly and annotation

Phage genomes were assembled using the Newbler assembly software package (454 Life Sciences, Branford, CT) with all settings set to default and the ‘-finish’ mode invoked. The ‘-finish’ mode assembles through repetitive regions that form unambiguous paths between contigs, thus some regions that would typically generate an assembly gap were assembled into a contig. Consensus genome sequences reported here represent from 11.9- to 23.8-fold coverage, depending upon the phage, with quality scores better than Q40 for > 99.3% of the bases (Henn *et al.*, 2010).

The assembled genomes were annotated in a pseudo-automated pipeline as follows. ORF predictions were made using GeneMarkS (Besemer *et al*., 2001), then manually refined based upon synteny and maximizing ORF size where alternate start sites were present. We next used all predicted ORFs from the 26 T4 phages as blastn queries against the genome sequences to pull out all possible ORFs (*e*-value cut-off < 1e-5). In this way, we identified a small number of cases (< 1%) where the ORF existed in a genome, but had not been predicted by GeneMarkS or manual annotation. Functional annotation to predicted ORFs were assigned using blastp (*e*-value cut-off < 1e-3) against the NCBI non-redundant database (as of April 2009) in combination with gene size and synteny information and HMM profiles for T4-GCs (described below) were HHsearched against the PFAM database. Identification of tRNA genes were done using tRNA-Scan-SE (Lowe and Eddy, 1997). Bacterial sigma-70 promoters and terminators were predicted using BPROM (LDF > 2.75, Softberry, Mount Kisco, NY) and TransTermHP (confidence score > 80% with an energy score of < −11 and a tail score of < −6; Kingsford *et al*., 2007), respectively, using default parameters. As well, we specifically searched for known T4 promoters and cyanobacterial nutrient-related promoters as follows. Early T4 phage promoters are sigma-70 promoters that are predicted from the BPROM analysis described above, while to determine T4 late promoters, the known T4 late promoter sequence 5′-TATAAAT-3′ (Miller, 2003a,b;) was used as a query on an initial blastn search (*e*-value cut-off < 10), over the entire genomes. The resulting sequences were use in a second blastn search (*e*-value cut-off < 10) to allow for mismatches and obtain further possible promoters. Then only those present in intergenic regions or 10 bp of overlap in the immediate upstream gene were used. Subsequently, known cyanobacterial Pho and *ntcA* promoters were identified using consensus sequences for known Pho boxes (5′-CTTAN7CTTA-3′; Su *et al*., 2007) and using the probabilistic model of *ntcA* binding sites (Su *et al*., 2005) that was more specifically adapted for use with marine cyanobacteria (5′-GTA-N8-TAC-3′; Su *et al*., 2006). In addition to probability scoring cut-offs, all promoters or terminators also were required to be intergenic or within 10 bp of the start/stop of an ORF.

The 12 new cyanophage genome annotations (GU071094-GU071099, GU071101, GU071103, GU071105-GU071106, GU071108, GU075905), and the four previously published cyanophage genome annotations (DQ149023, AJ630128, AY940168, AY939844, FM207411) are available at GenBank, while the 10 non-cyanophage genome annotations are available at http://phage.bioc.tulane.edu. Additionally, all 26 T4-like phage genome GenBank accession numbers are available in Table 1, and all 16 new or updated cyanophage genomes are also available as a single project at the CAMERA database (http://web.camera.calit2.net/cameraweb/gwt/org.jcvi.camera.web.gwt.download.ProjectSamplesPage/ProjectSamplesPage.oa?projectSymbol=CAM_PROJ_BroadPhageGenomes).

Whole-genome sequencing of these phages revealed that three previously published gene sequences derived from PCR products from these phages (Sullivan *et al*., 2006; 2008;) were incorrect: *g20* from Syn33 (gene GI:189397306, protein GI:189397307), *g20* from S-SSM7 (gene GI:189397276, protein GI:189397277) and *psbA* from S-SSM5 (gene GI:95115381, protein GI:95115382). These previous GenBank accessions for these sequences have been corrected with the sequences from the genomes.

### Protein clustering and divergent sequence annotation

The method for clustering orthologous genes across the 26 T4-like phage genomes was similar to that described previously (Kettler *et al*., 2007). Briefly, pairwise orthologous relationships were mapped in all T4-like genomes using reciprocal best blastp hit (*e*-value ≤ 1e-5) to each other where the sequence alignment length was at least 75% of the protein length of the shorter gene of the two compared. T4 Gene Clusters (T4-GCs) were then built by transitively clustering these orthologues together, where if gene A and B are orthologues and gene B and C are orthologues, then genes A, B and C are clustered into an orthologous group. To find divergent orthologues missed by the initial blast-based approach, we built HMM profiles (Durbin *et al*., 1998) for the T4-GCs, and then searched singleton T4 genes that were not grouped into any T4-GC against the T4-GC HMM profiles. T4-GC HMM profiles were built by aligning each gene in a T4-GC using muscle version 3.7 (Edgar, 2004) with default parameters and then using hmmbuild from hmmer version 2.3.2 (http://hmmer.janelia.org/) to build the HMM profiles from the resulting alignments. The program hmmsearch also from the hmmer version 2.3.2 was used to search a protein sequence against these in-house T4-GC HMM profiles. Those singletons with significant homology (*e*-value ≤ 1e-5) to T4-GC HMMs were considered for membership in that T4-GC and manually curated to certify membership. A total of 15 single genes were brought into T4-GCs this way.

A multifasta of all ORFs used in this study is provided as a supplementary file which includes in the fasta header the ORF identifier and genome location, T4-GC assignment and functional annotation (File S2).

### Gene presence/absence network analysis

A presence/absence table of all T4-GCs in the 26 phage genomes was constructed and displayed as a network using the spring-embedded layout option Cytoscape 2.5 (Fig. 2) (Cline *et al*., 2007). This layout option treats the connections (edges) between nodes as springs that repel or attract nodes to each other according to a force function; nodes are positioned to minimize the sum of forces in the network. Nodes in the graph represent the T4-GCs (circles) and the genomes (diamonds), and edges represent the presence of a particular T4-GC in a given genome. Each genome node will therefore have a set of T4-GC nodes connected to it. The resulting network highlights the similarities between genomes based on the presence and absence of gene clusters in each genome.

### Virion structural proteomics

Structural proteomic experiments were conducted as described previously (Sullivan *et al*., 2009). Briefly, the samples were incubated in a denaturing solution of 8 M Urea/1% SDS/100 mM ammonium bicarbonate/10 mM DTT pH 8.5 at 37°C for 1 h. Next, the samples were alkylated for 1 h by the addition of iodoacetamide to a final concentration of 40 mM and then quenched with 2 M DTT. Following the addition of 4× LDS loading buffer (Invitrogen), each sample was centrifuged at 14 000 r.p.m. for 5 min at room temperature, and each sample was fractionated on a NuPAGE 10% Bis-Tris 10 lane gel (Invitrogen) for 2.5 h at 125 volts, 50 mA and 8 W. Gels were shrunk overnight by the addition of 50% ethanol and 7% acetic acid, and then allowed to swell for 1 h by the addition of deionized water. Gels were stained with SimplyBlue Safe Stain (Invitrogen) for 2–4 h, imaged, and sliced horizontally into fragments of equal size based on the molecular weight markers.

In-gel digestion was performed after destaining and rinsing the gel sections with two washes of 50% ethanol and 7% acetic acid, followed by two alternating washes with 50 mM ammonium bicarbonate and acetonitrile. After removal of the last acetonitrile wash, 100 µl of sequencing grade trypsin (Promega) was added to each gel slice at a concentration of 6.6 ng µl^−1^ in 50 mM ammonium bicarbonate/10% acetonitrile. The gel slices were allowed to swell for 30 min on ice, after which the tubes were incubated at 37°C for 24 h. Peptides were extracted with one wash of 100 µl of 50 mM ammonium bicarbonate/10% acetonitrile and one wash of 100 µl of 50% acetonitrile/0.1% formic acid. The extracts were pooled and frozen at −80°C, lyophilized to dryness and redissolved in 40 µl of 5% acetonitrile, 0.1% formic acid.

Samples were then loaded into a 96-well plate (AbGene) for mass spectrometry analysis on a Thermo Fisher Scientific LTQ-FT. For each run, 10 µl of each reconstituted sample was injected with a Famos Autosampler, and the separation was performed on a 75 mM × 20 cm column packed with C_18_ Magic media (Michrom Biosciences) running at 250 nl min^−1^ provided from a Surveyor MS pump with a flow splitter with a gradient of 5–60% water, 0.1% formic acid, acetonitrile 0.1% formic acid over the course of 120 min (150 min total run). Between each set of samples, standards from a mixture of five angiotensin peptides (Michrom Biosciences) were run for 2.5 h to ascertain column performance and observe any potential carryover that might have occurred. The LTQ-FT was run in a top five configuration with one MS 200 K resolution full scan and five MS/MS scans. Dynamic exclusion was set to one with a limit of 180 s with early expiration set to two full scans.

Peptide identifications were made using sequest (ThermoFisher Scientific) through the Bioworks Browser 3.3. The data were searched with a 10 ppm window on the MS precursor with 0.5 Da on the fragment ions with no enzyme specificity. A reverse database strategy (Elias and Gygi, 2007) was employed with a six-frame translation of the genomic sequence reversed and concatenated with the forward sequences supplemented with common contaminates and filtered to obtain a false discovery rate of less than or equal to 1%. Peptides passing the filters were mapped back onto the genome and compared with predicted ORFs.

## Note added in proof

After we completed the analyses of the cyanophage genomes described in this article, another *Synechococcus* phage genome (S-RSM4) became available (Millard *et al*., 2009). The S-RSM4 genome appears to be a ‘standard *Synechococcus* T4 phage’ as inferred from its genome-wide %G+C (41%) and gene content (contains all 38 T4 core genes, all 25 cyano T4 core genes, all 12 nearly cyano T4 core genes, 21 *Synechococcus*-enriched genes and none of the *Prochlorococcus*-enriched genes).

Both S-RSM4 (Millard *et al*., 2009) and P-RSM5 (this study) were isolated from the oligotrophic Red Sea, and both contain a notable phosphate-related feature. Specifically, P-RSM5, which contains *pstS*, was isolated in September, after months of summer stratification (Lindell and Post, 1995; Fuller *et al*., 2005), which would dramatically reduce nutrient concentrations in surface waters. In contrast, S-RSM4, which lacks *pstS*, was isolated in April before summer stratification (Lindell and Post, 1995; Fuller *et al*., 2005), likely resulting in less stressful nutrient limitation. In fact, cyanobacterial *pstS* expression from these same waters was minimal (Fuller *et al*., 2005), consistent with a lack of phosphate stress in these waters. We hypothesize therefore that the presence/absence of *pstS* in these two phages also reflects the nutrient status of the waters from which they were collected.

## Supporting information

**Fig. S1**. Cyanophage genome size plotted as a function of the number of predicted ORFs where original host genera are designated by colour.

**Fig. S2**. The genome location of four hierarchical ‘core’ gene sets plotted for 26 T4 phage genomes. Lines connect function-based orthologues across genomes, and are coloured as per legend.

**Fig. S3**. Multiple sequence alignment of the T4 phage gp51 baseplate hub catalyst protein from 26 T4 phage genomes. The cyanophage and marine vibriophage copies of gp51 are significantly reduced, missing the first ∼200 amino acids relative to the non-cyano non-marine T4 phage copies (the first 140 amino acids of the alignment are not shown). In spite of this size difference, there is marked similarity in the C-terminal region of the protein shown in the alignment.

**Fig. S4**. Maximum likelihood tree of the pyrophosphatase MazG protein. The tree was constructed from 271 aligned amino acids, using PhyML and the JTT model of substitution with gamma-distributed rates empirically estimated from the data. The accession numbers for the sequences used in this analysis are available upon request. Numbers at the nodes represent bootstrap values for 1000 replicates.

**Fig. S5**. Weblogo (http://weblogo.berkeley.edu/) diagrams of the various bioinformatically predicted promoters in the cyanophage genomes.

**Fig. S6**. Multiple sequence alignment of the cyanophageencoded Zwf proteins identified in varying degrees of preservation across eight cyanophages. While the sequence conservation is minimal for the three highly degraded copies, their position in the genomes is conserved and remnants of sequence similarity remain along the protein.

**Fig. S7**. Alignment of the endonucleases in T4-GCs 228 and 282.

A. Putative homing endonucleases (T4-GC282) where only the P-SSM2 copy has conserved catalytic residues as compared with the experimentally characterized homing endonuclease present in S-PM2 (S-PM2p177, Zeng et al., 2009). The remaining copies appear to have lost these residues and are likely non-functional, yet are all located at a conserved region suggesting a single evolutionary event of insertion at the 3′-end of gp17 (see upper panel for genome sequence details).

B. Possible endonucleases (T4-GC228) which lack the conserved residues in (A) but nonetheless are highly conserved and proximal to the carbon metabolism genes, suggesting that they may be responsible for genetic shuffling in this region.

**Fig. S8**. Close-up genome representation of the mobile carbon metabolic gene cluster from cyanophage genomes. Genomic features are as described in Fig. 1, and genome location orientation is as described for Fig. 5.

**Fig. S9**. Close-up genome representation of the mobile hypothetical genes cluster from cyanophage genomes. Genomic features are as described in Fig. 1, and genome location orientation is as described for Fig. 5.

**Table S1**. Detailed features of the T4-like ocean cyanophage isolates.

**Table S2**. T4-like phage core genes determined from 16 cyanophages and 10 non-cyanophages. Numbers listed for each phage represent the size of the genes (bp), with multiple copies separated by a ‘|’. Some T4-GCs were pooled to create a single functional category based upon annotation and genome synteny.

**Table S3**. Non-cyano T4-like ‘core’ beyond the T4-core. Numbers listed for each phage are as in Table S2.

**Table S4**. Cyano T4-like core genes. Numbers listed for each phage are as in Table S2.

**Table S5**. Proteins that are unique to either P-HM1 or P-HM2 phage genome in pairwise comparison of these two co-isolated phages.

**Table S6**. *Synechococcus* phage-enriched proteins. Numbers listed for each phage are as in Table S2.

**Table S7**. *Prochlorococcus* phage-enriched proteins. Numbers listed for each phage are as in Table S2.

**Table S8**. Summary of cyano T4 proteomics experiments. Comparative proteomics = experimentally determined protein content in purified virus particles to determine the structural proteins in three sequenced T4-like virus genomes. A ‘Y’ means the protein was detected, ‘–’ means the protein is annotated in the genome but no peptides were detected, ‘NP’ means the protein is not present in the genome, ‘counts’ are the number of peptide fragments detected per protein, ‘copy # in T4’ refers to the biochemically and ultrastructurally determined copy number of proteins in the coliphage T4 particle. Ten of these proteins, in italics, have similar distributions among nine cyanophages and may be functionally linked.

**File S1**. The spreadsheet used to generate the overview of the cyanophage genome annotations that are presented in Fig. 1.

**File S2**. Multifasta of all ORFs examined in this study including gene identifiers and genome location, T4-GC assignment and functional annotation.
