# Supplementary material for: Genomic analysis of oceanic cyanobacterial myoviruses compared with T4-like myoviruses from diverse hosts and environments
Source: Environ Microbiol. 2010 Nov;12(11):3035–56. doi: 10.1111/j.1462-2920.2010.02280.x (PMC3037559; doi:10.1111/j.1462-2920.2010.02280.x)
Supplement: Supplementary file 1 [file emi0012-3035-SD1.ppt]

## Slide 1
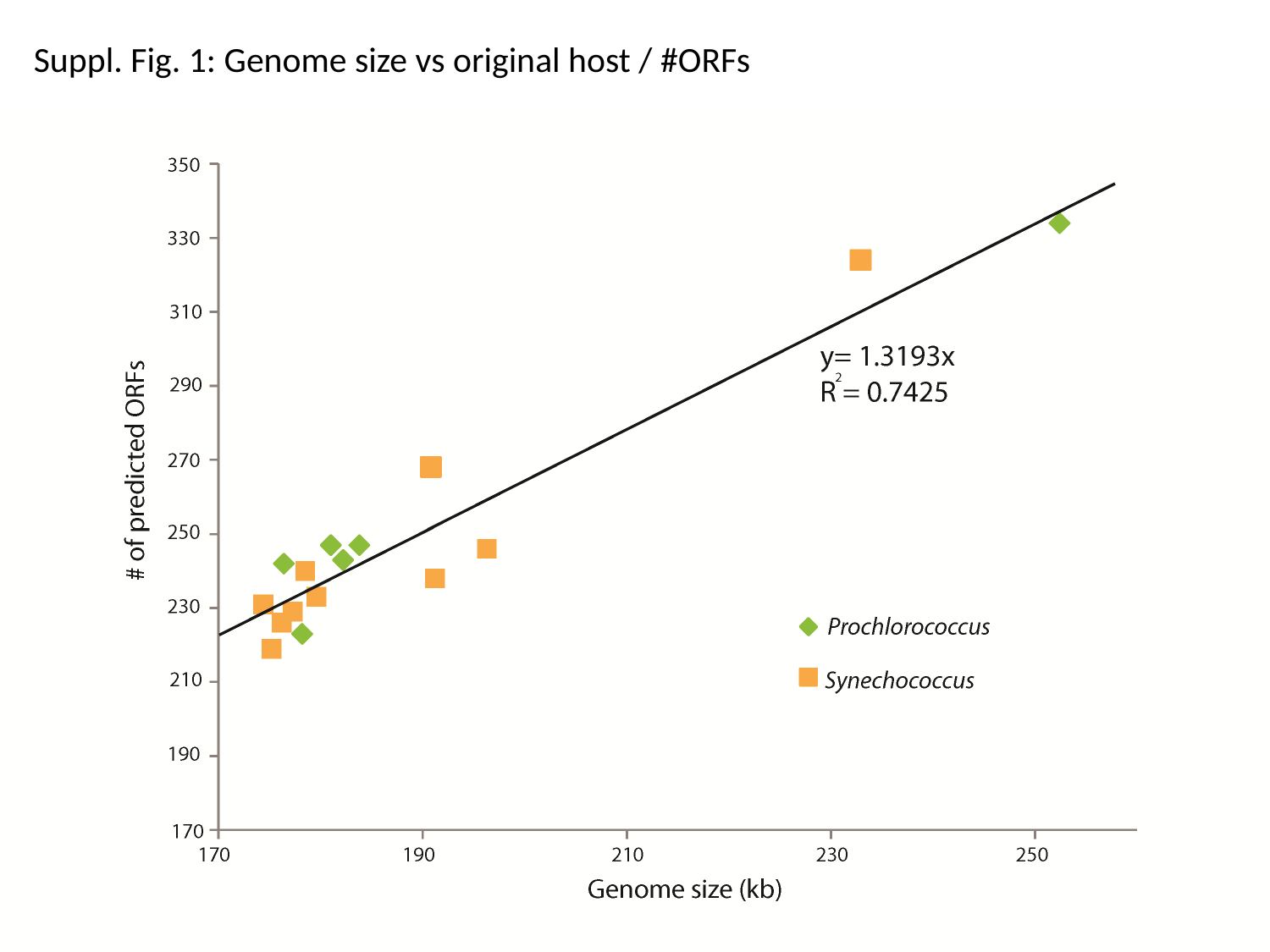

# Suppl. Fig. 1: Genome size vs original host / #ORFs

## Slide 2
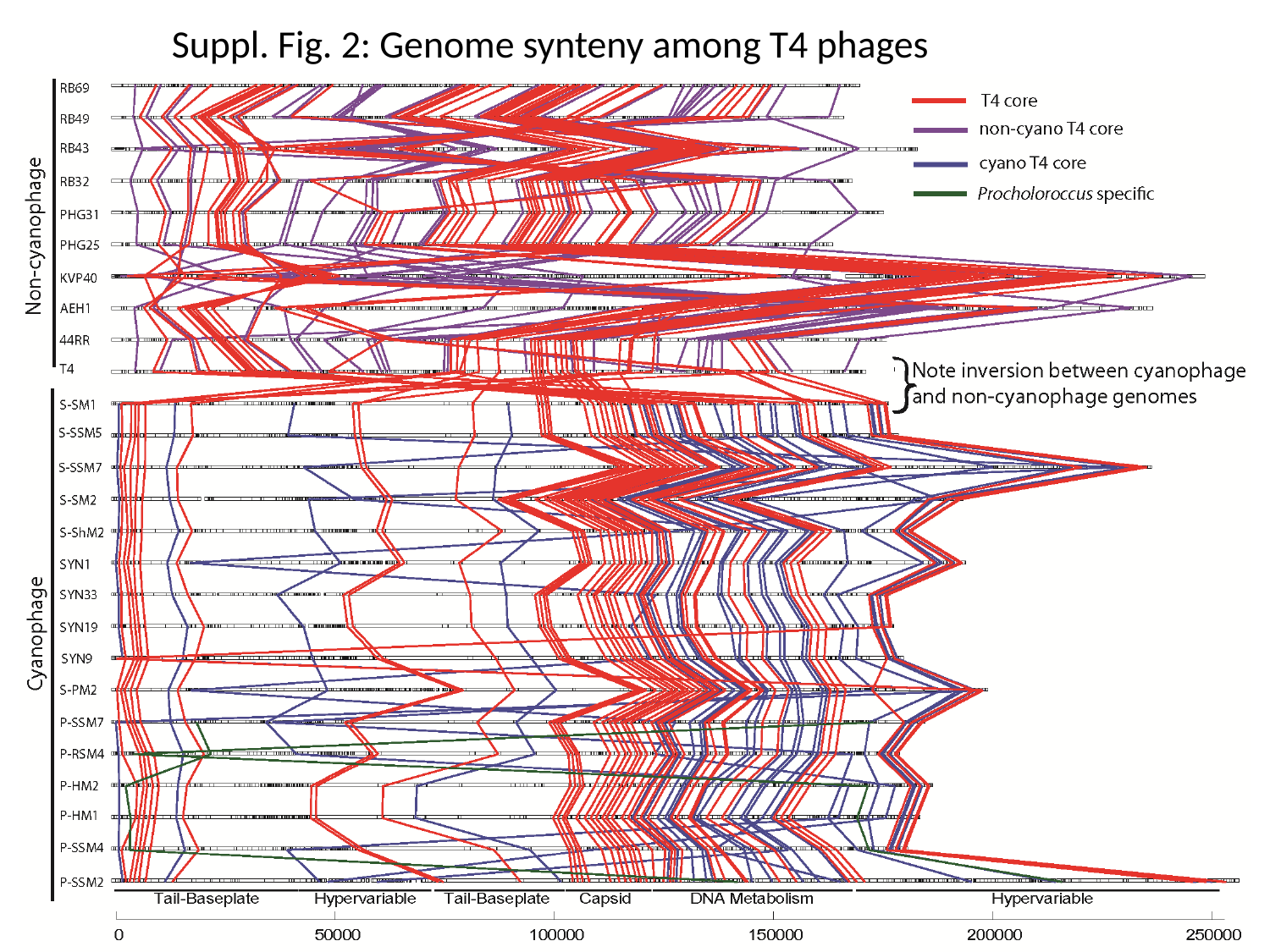

# Suppl. Fig. 2: Genome synteny among T4 phages

## Slide 3
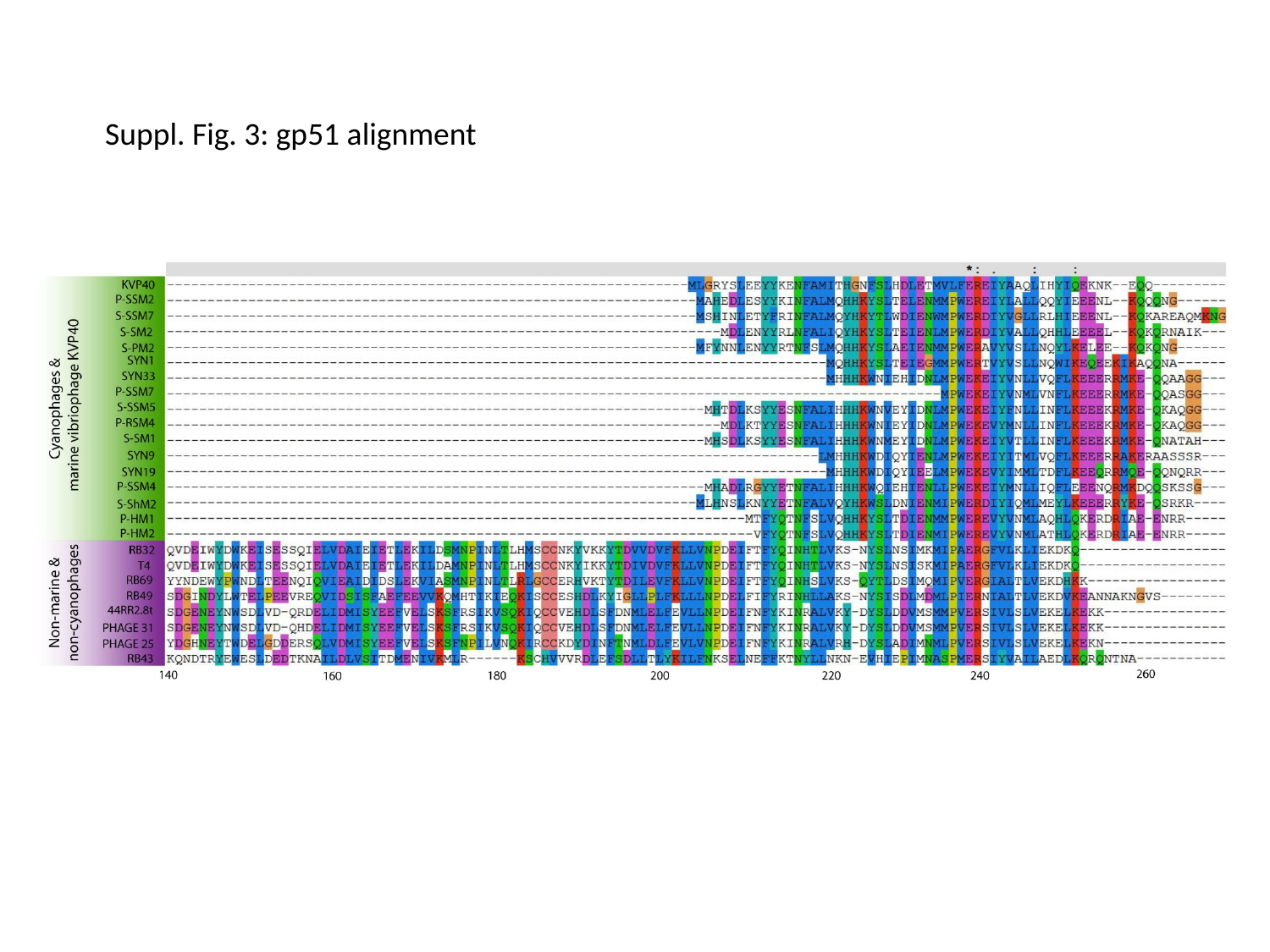

# Suppl. Fig. 3: gp51 alignment

## Slide 4
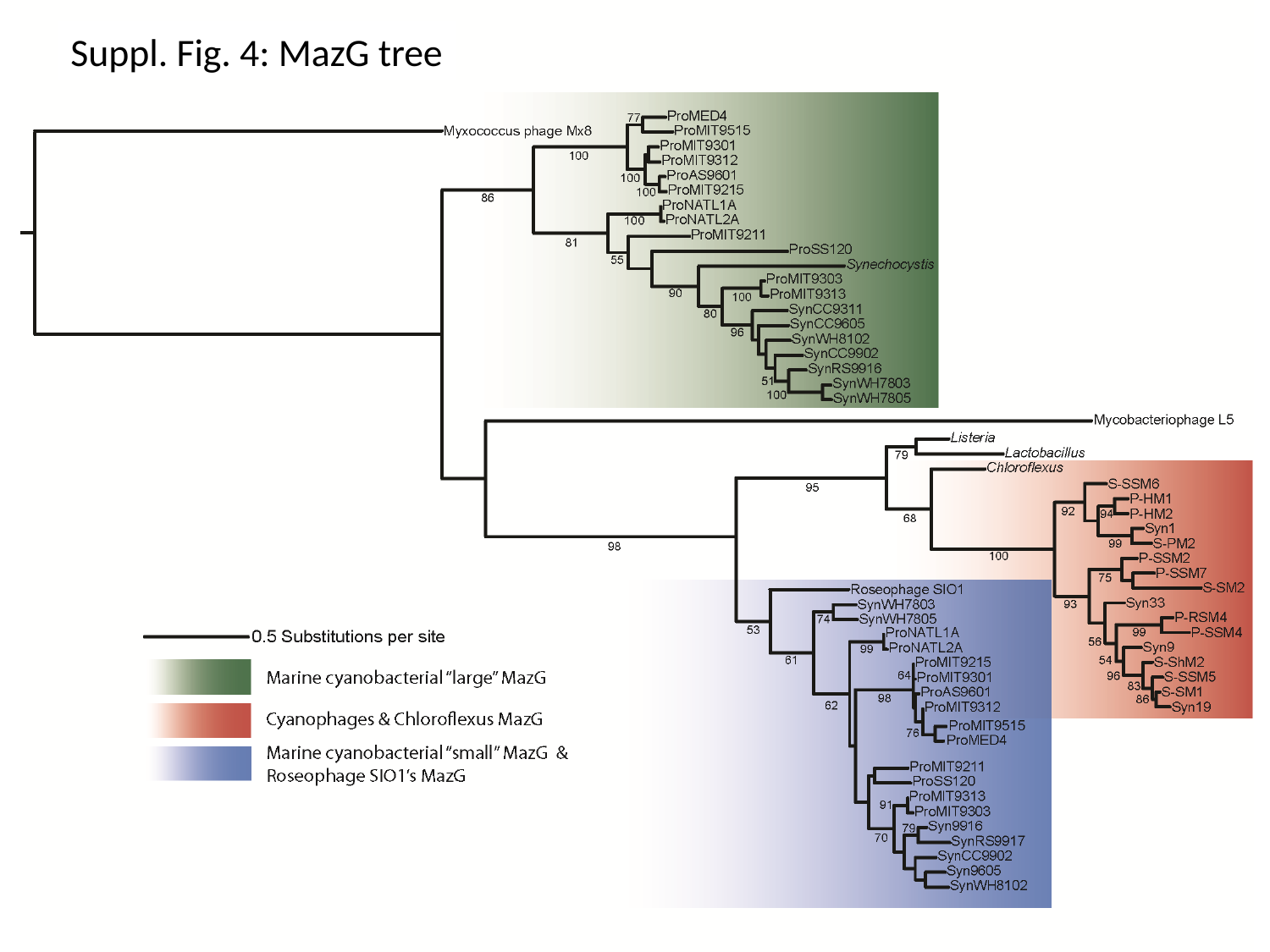

Suppl. Fig. 4: MazG tree

## Slide 5
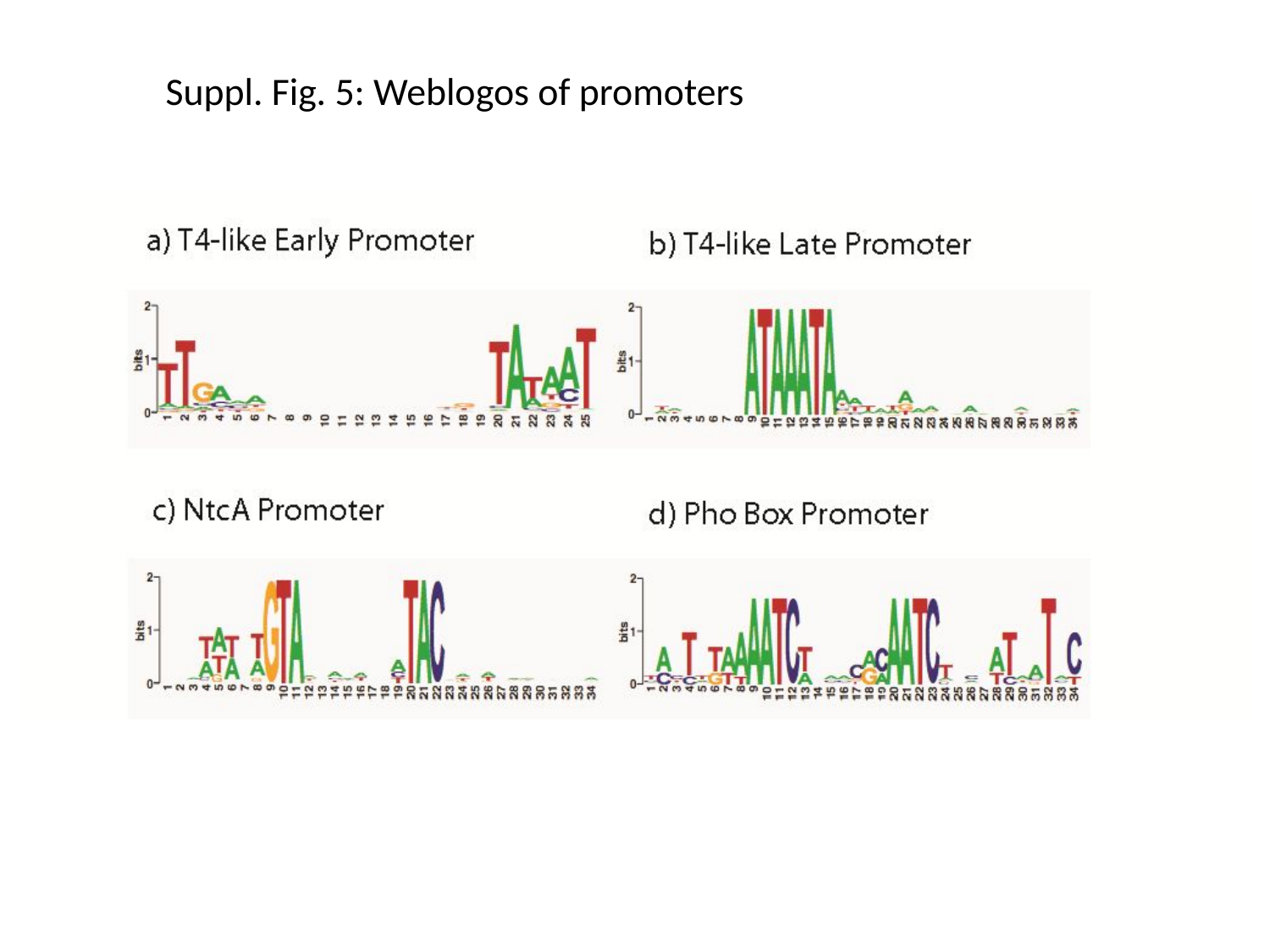

# Suppl. Fig. 5: Weblogos of promoters

## Slide 6
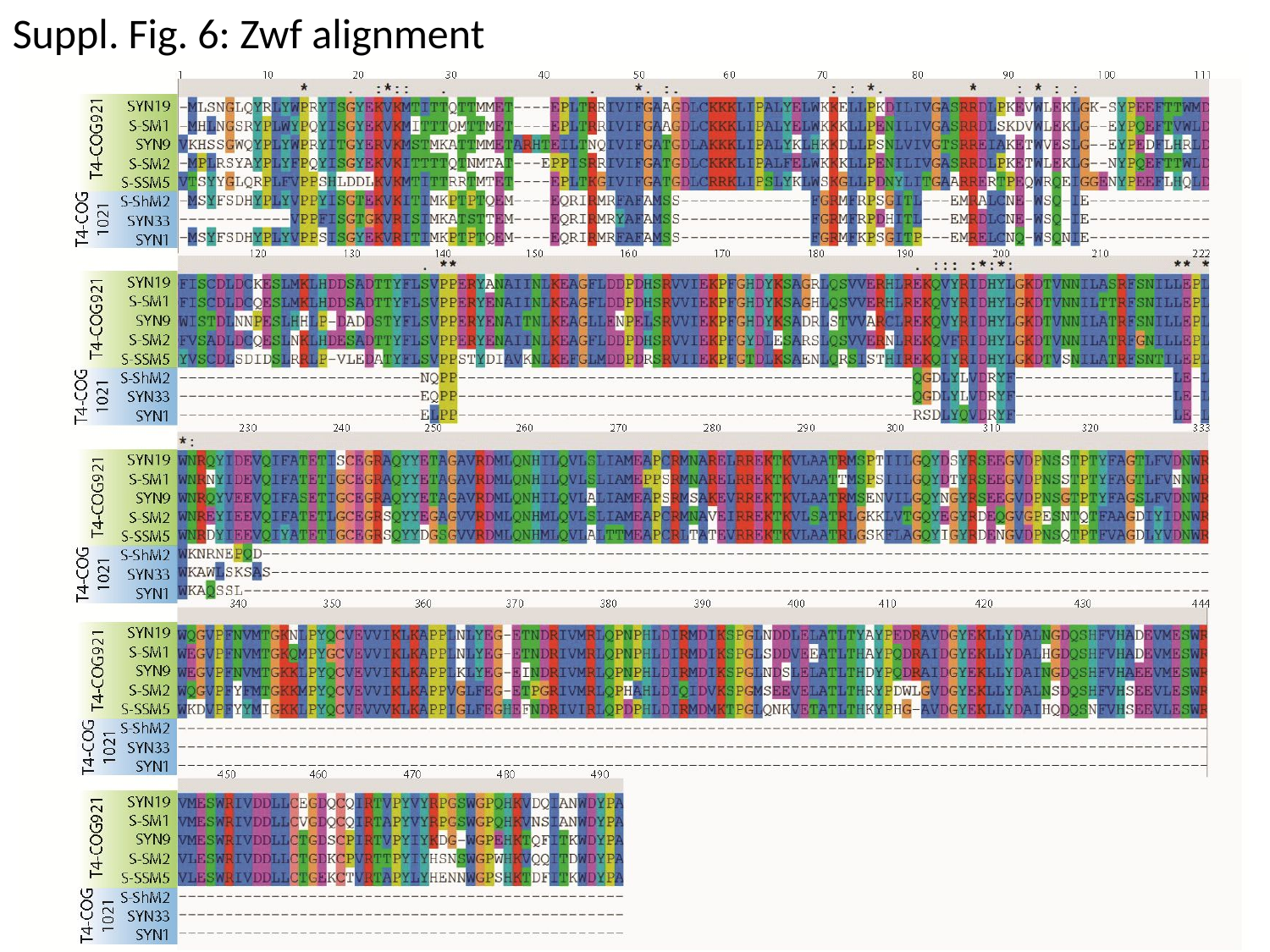

# Suppl. Fig. 6: Zwf alignment

## Slide 7
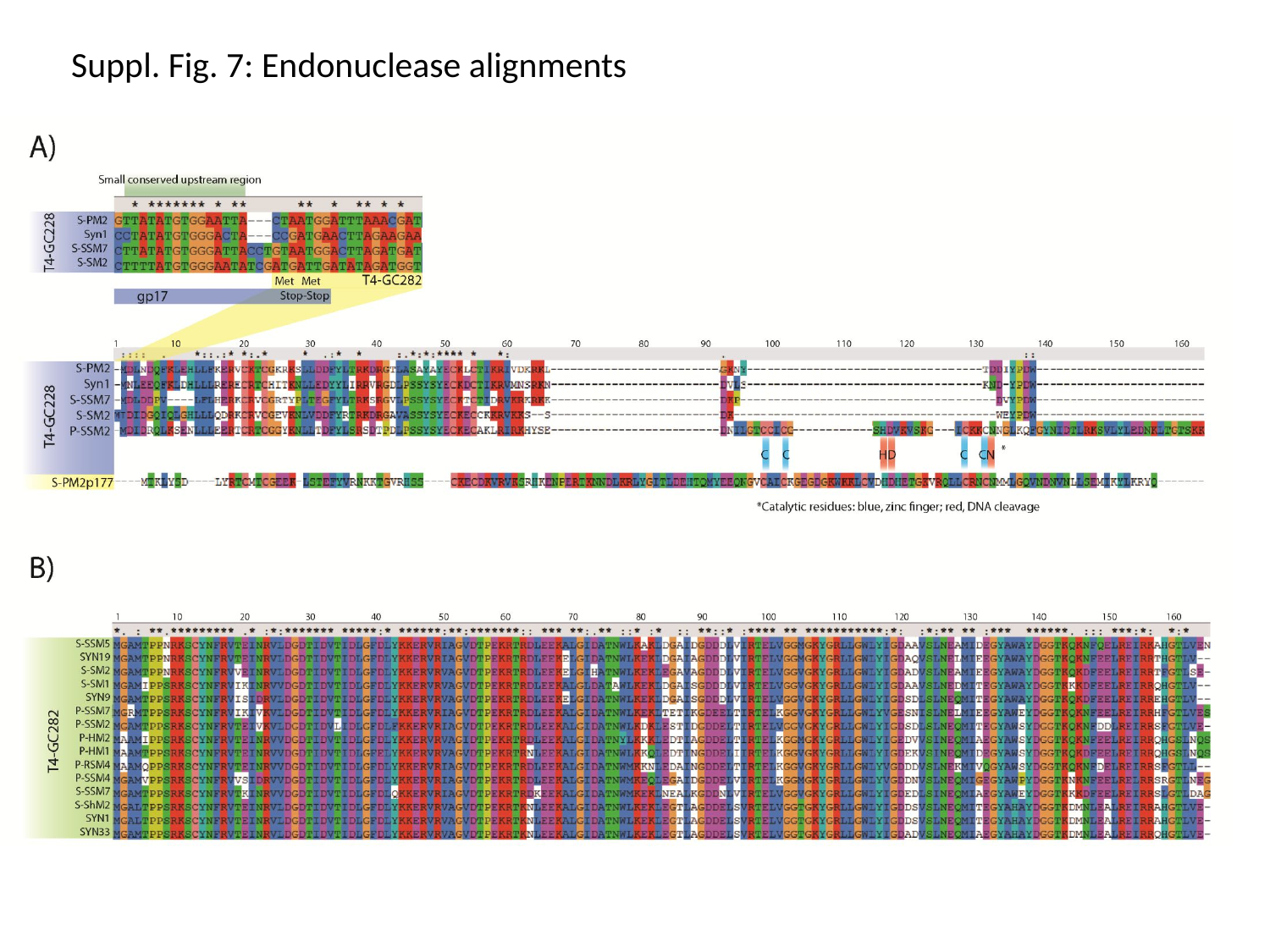

# Suppl. Fig. 7: Endonuclease alignments

## Slide 8
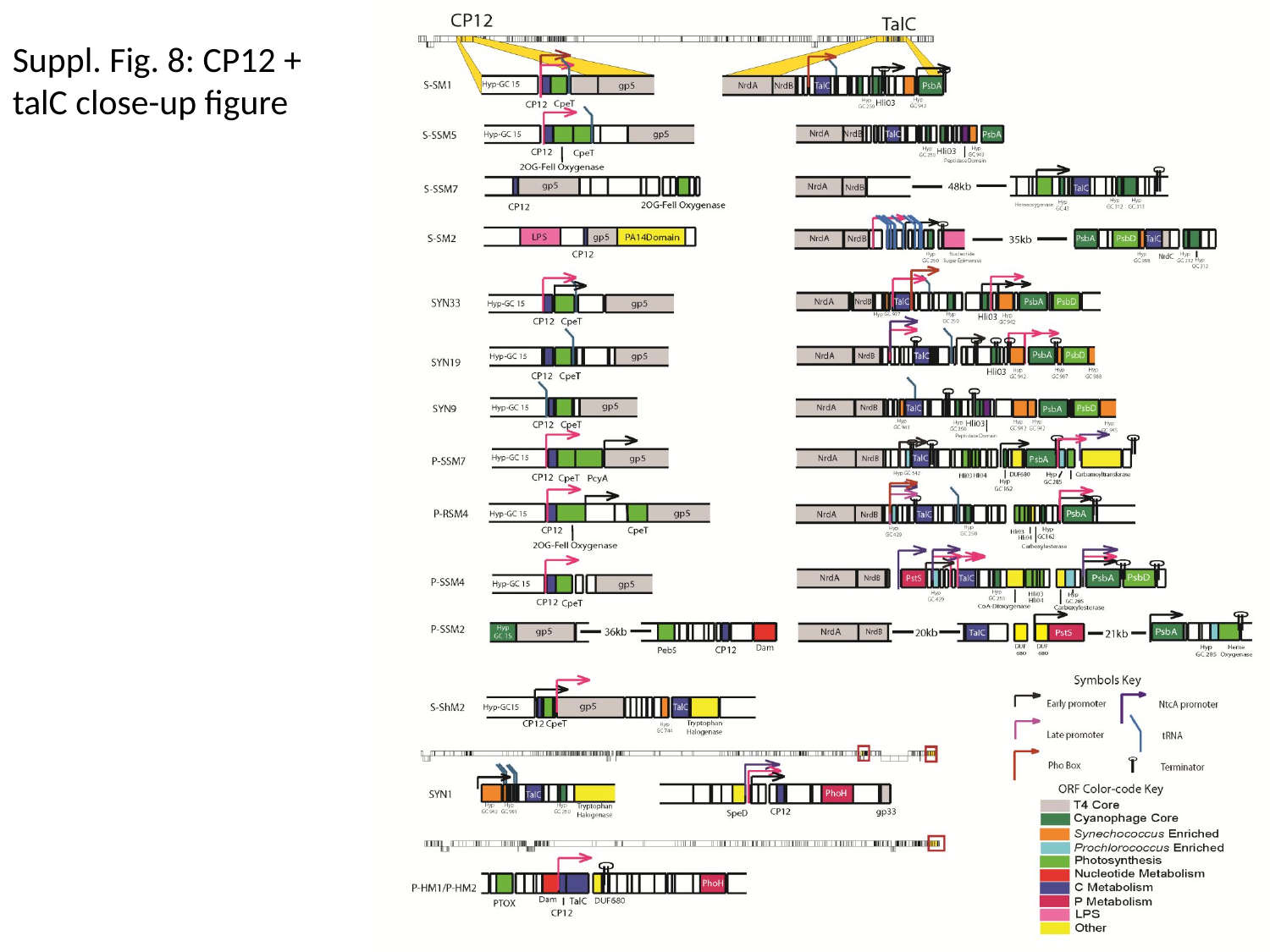

# Suppl. Fig. 8: CP12 + talC close-up figure

## Slide 9
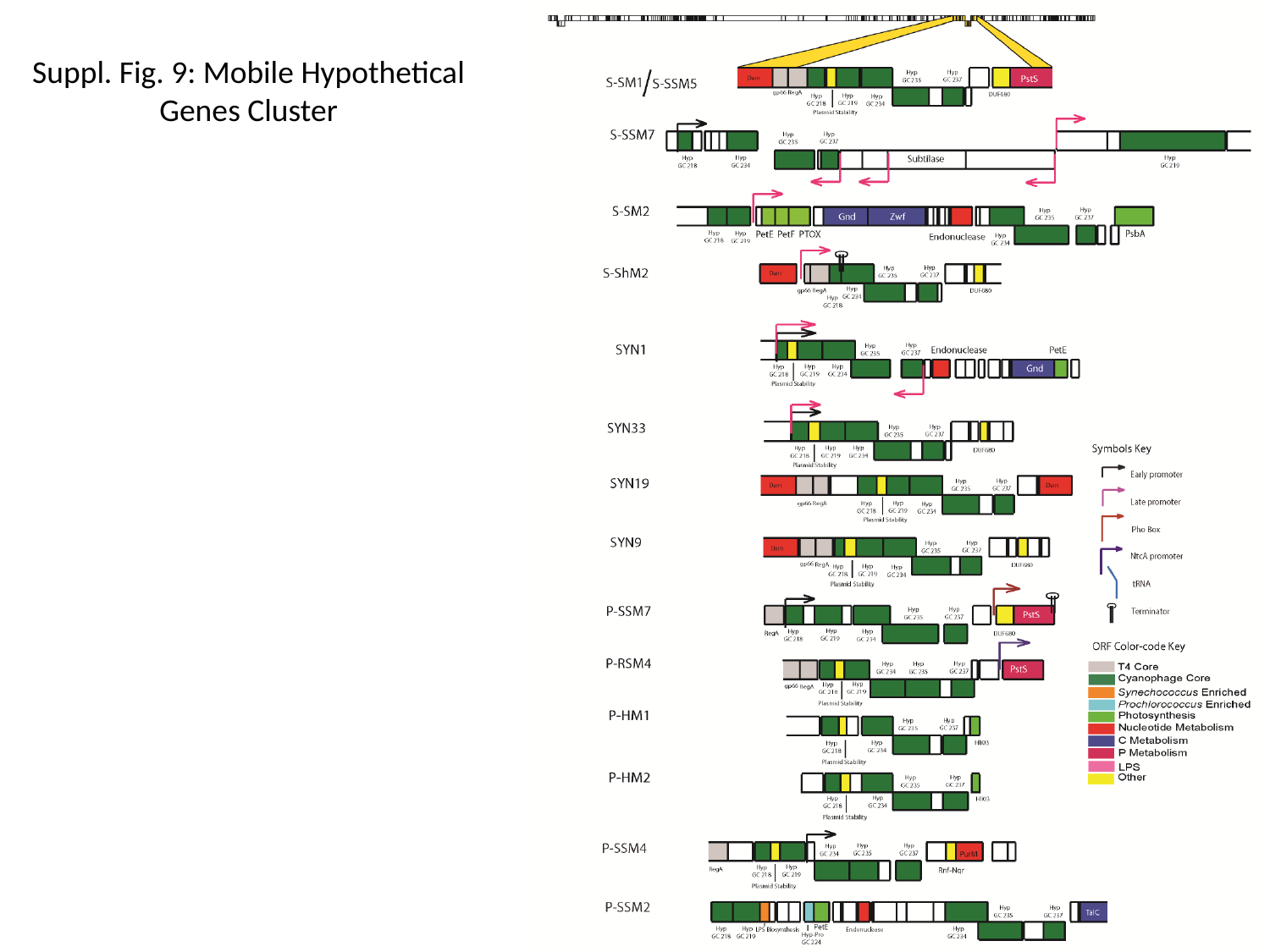

# Suppl. Fig. 9: Mobile Hypothetical Genes Cluster
