# Supplementary material for: Genomic analysis of oceanic cyanobacterial myoviruses compared with T4-like myoviruses from diverse hosts and environments
Source: Environ Microbiol. 2010 Nov;12(11):3035–56. doi: 10.1111/j.1462-2920.2010.02280.x (PMC3037559; doi:10.1111/j.1462-2920.2010.02280.x)
Supplement: Supplementary file 2 [file emi0012-3035-SD2.pdf]

Suppl. Table 1: Detailed features of the T4-like ocean cyanophage isolates

Published name	Original host*	Size (kb)	# ORFs	%G+C	Source water details	Date water sampled	Temp ('C)	Salinity (ppt)	P (umol/kg)	NO3+NO2 (umol/kg)	# tRNA	tRNA's	Ref
**Cyanophages**													
P-SSM2	ProNATL1A	252.4	330	35.5%	31°48'N, 64°16'W, BATS, 100m	6-Jun-00	19.0	36.7	N.A.	N.A.	1	Asn (AAC);	1
P-SSM4	ProNATL2A	178.2	198	36.7%	31°48'N, 64°16'W, BATS, 10m	6-Jun-00	26.0	36.4	N.A.	N.A.	0	---	1
P-HM1	ProMED4	181	247	38.0%	22°45'N, 158°00'W, Station ALOHA, 125m	9-Mar-06	22.7	35.3	0.05	0.01-0.45	0	---	2
P-HM2	ProMED4	183.8	248	38.0%	22°45'N, 158°00'W, Station ALOHA, 125m	9-Mar-06	22.7	35.3	0.05	0.01-0.45	0	---	2
P-RSM4	ProMIT9303	176.4	246	38.0%	29°28'N, 34°53'E, Red Sea 130m	13-Sep-00	22.0	41.0	N.A.	N.A.	3	Leu (TTA); Arg (AGA); Met (ATG);	2
P-SSM7	ProNATL1A	182.2	241	37.0%	31°48'N, 64°16'W, BATS, 120m	Sep-99	20.3	36.7	N.A.	N.A.	4	Leu (TTA); Arg (AGA); Asn (AAC); Ile (ATA);	2
S-PM2	SynWH7803	196.3	238	37.8%	50°18'N, 4°12'W, English Channel, 0m	23-Sep-92	N.A.	N.A.	N.A.	N.A.	24	Met (ATG) X3; Leu (TTA); Leu (CTA); Arg (AGA); Asn (AAC); Val (GTA); Thr (ACA); Ala (GCA); Gly (GGA); Ile (ATA); Ser (AGC); Ser (TCC); Pro (CCA); Lys (AAA); Tyr (TAC); Asp (GAC); Glu (GAA); Ile (ATC); His (CAC); Gln (CAA); Trp (TGG); Arg(CGT);	3
Syn9	SynWH8109	177.3	235	40.50%	41°31'N, 71°40'W, Woods Hole, 0m	Oct-90	15.0	N.A.	N.A.	N.A.	6	Leu (TTA); Arg (AGA); Asn (AAC); Val (GTA); Thr (ACA); Ala (GCA);	4
Syn19	SynWH8109	175.2	229	41.0%	34°06'N, 61°01'W, Sargasso Sea, 0m	Jul-90	26.5	N.A.	N.A.	N.A.	6	Leu (TTA); Arg (AGA); Asn (AAC); Val (GTA); Thr (ACA); Ala (GCA);	2
Syn33	SynWH7803	174.4	238	40.0%	25°51'N, 79°26'W, Gulf Stream, 0m	Jan-95		N.A.	N.A.	N.A.	5	Leu (TTA); Arg (AGA); Asn (AAC); Val (GTA); Thr (ACA);	2
Syn1	SynWH8101	191.2	224	41.0%	41°31'N, 71°40'W, Woods Hole, 0m	Aug-90	23.0	N.A.	N.A.	N.A.	6	Leu (CTA); Arg (AGA); Asn (AAC); Val (GTA); Thr (ACA); Gly (GGA);	2
S-ShM2	SynWH8102	179.6	231	41.0%	39°60'N, 71°48'W, Atlantic Shelf Waters, 0m	16-Sep-01	20.7	33.4	N.A.	0.043	1	Arg (AGA);	2
S-SM2	SynWH8017	190.8	292	40.0%	38°10'N, 73°09'W, Atlantic Slope Waters, 15m	17-Sep-01	24.0	35.9	N.A.	0.049	10	Leu (TTA); Arg (AGA); Asn (AAC); Val (GTA); Thr (ACA); Ala (GCA); Gly (GGA); Ile (ATA); Ser (TCA); Pro (CCA);	2
S-SSM7	SynWH8109	232.9	324	39.0%	34°24'N, 72°03'W, W Sargasso Sea, 70m or 95m	22-Sep-01	22.0	36.8	N.A.	N.A.	5	Leu (TTA); Arg (AGA); Ile (ATA); Thr (ACA); Gly (GGA);	2
S-SSM5	SynWH8102	176.2	229	40.0%	34°24'N, 72°03'W, W Sargasso Sea, 70m	22-Sep-01	23.7	36.7	N.A.	N.A.	4	Leu (TTA); Arg (AGA); Val (GTA); Thr (ACA);	2
S-SM1	SynWH6501	178.5	239	41.0%	38°10'N, 73°09'W, Atlantic Slope Waters, 0m	17-Sep-01	24.0	35.9	N.A.	0.011	6	Leu (TTA); Arg (AGA); Asn (AAC); Val (GTA); Thr (ACA); Ala (GCA);	2

* original hosts are either genus *Prochlorococcus* indicated by "Pro" or *Synechococcus* indicated by "Syn"

References: 1 = Sullivan et al. 2005, 2 = this study, 3 = Mann et al. 2007, 4 = Weigele et al. 2007

N.A. = data not available

Suppl. Table 2: T4-like phage core genes determined from 16 cyanophages and 10 non-cyanophages***. Numbers listed for each phage represent the size of the genes (bp), with multiple copies separated by a "|". Some T4-GCs were pooled to create a single functional category based upon annotation and genome synteny.

		cyanophages																non-cyanophages									
T4-GC #	GENE DESCRIPTION	P-SSM2	P-SSM4	P-HM1	P-HM2	P-RSM4	P-SSM7	S-PM2	Syn9	Syn19	Syn33	Syn1	S-ShM2	S-SM2	S-SSM7	S-SSM5	S-SM1	T4	44RR	Aeh1	KVP40	PHG25	PHG31	RB32	RB43	RB49	RB69
133	gp3 head-proximal tip of tail tube tail completion + sheath stabilizer protein	534	552	558	546	549	549	510	573	570	549	506	567	576	561	549	549	531	528	570	534	528	528	531	534	591	585
9	gp4 head completion protein	438	426	435	462	426	420	438	420	420	420	342	438	444	480	426	474	453	453	471	456	453	453	453	435	474	450
340+455+16+ 1156+2374	gp5 baseplate hub + tail lysozyme	2259	2310	1764|870	2787|873	2541	2553	2946	2508	2385	2580	2922	2484	1848	3027	2544	2553	1728	1803	1815	1266	1674	1803	1728	1770	1803	1734
106	gp6 baseplate wedge	1944	1989	1851	1851	2028	2031	1809	2031	2025	2031	1809	2133	2112	1875	2028	2019	1983	1884	1956	1959	1884	1884	1983	1854	1905	1971
108+1594	gp8 baseplate wedge	1602	1533	1515	1515	1539	1533	1905	1533	1533	1533	1884	1533	1551	1596	1533	1533	1005	987	987	1032	987	987	1005	993	996	1005
116	gp13 neck protein	846	807	816	816	810	807	831	807	807	807	831	807	846	819	807	807	930	924	921	924	945	924	930	924	933	927
117	gp14 neck protein	1413	927	1329	1329	933	927	879	1173	921	1173	879	1164	1455	2289	927	927	771	759	789	837	762	759	771	747	741	765
118	gp15 proximal tail sheath stabilization	843	1032	1005	1005	786	789	801	789	1032	789	801	837	1002	1083	786	786	819	819	828	1353	822	819	819	741	834	777
120	gp16 terminase DNA packaging enzyme, small subunit	432	435	489	411	432	432	420	432	429	432	426	405	414	438	432	432	495	465	519	534	450	465	495	537	498	495
124	gp17 terminase DNA packaging enzyme large subunit	1644	1653	1683	1683	1644	1653	1647	1650	1650	1650	1653	1089	1650	1659	1650	1650	1833	1842	1902	1803	1839	1842	1833	1830	1824	1836
125	gp18 tail sheath monomer	2190	2250	2010	2034	2253	2250	2232	2262	2259	2253	1908	1413	2247	2421	2253	2250	1980	1992	2040	2016	1992	1992	1980	1995	2001	1983
126	gp19 tail tube monomer	588	591	615	615	585	588	615	609	558	579	618	708	612	684	585	588	492	489	489	501	492	489	492	486	495	492
127	gp20 portal vertex protein of head	1677	1614	1671	1683	1602	1614	1695	1602	1596	1335	1680	1650	1668	1692	1605	1602	1575	1551	1566	1536	1551	1551	1575	1575	1566	1572
129	gp21 prohead core scaffold and protease	651	645	648	648	645	645	645	645	723	645	645	645	651	735	645	645	639|573	633	630	642	633	633	654	648	696	642
408+130	gp22 scaffoldprohead core protein	1101	1002	1053	1053	1047	1050	1179	1038	1026	1041	1185	1131	1098	1089	1038	1020	810	825	795	843	831	825	810	789	795	813
131	gp23 precursor of major head subunit	1413	1389	1368	1368	1398	1389	1407	1374	1374	1380	1407	1398	1404	1407	1395	1377	1566	1590	1605	1545	1590	1590	1566	1575	1587	1569
105	gp25 base plate wedge subunit	402	420	390	396	420	420	393	417	420	420	393	438	402	417	420	420	399	384	423	420	384	384	399	393	387	399
11	gp26 baseplate hub subunit	720	711	699	699	717	708	717	723	714	711	717	720	720	717	711	711	627	615	777	849	606	615	627	558	630	627
5	gp32 ssDNA binding protein	819	924	909	876	921	927	888	948	1026	930	873	999	966	921	981	1020	906	888	909	909	885	888	909	981	969	900
326+2112+ 2096+1667	gp33 late promoter transcription factor	261	261	351	351	390	252	249	249	306	309	249	306	258	336	306	306	339	258	231	297	255	258	339	252	270	339
182	gp41 DNA primase-helicase	1383	1179	1374	1368	1380	1371	1413	1389	1380	1458	1404	1380	1377	1386	1380	1410	1428	1410	1458	1401	1350	1407	1428	1437	1413	1443
178	gp43 DNA polymerase	2496	2481	2487	2487	2487	2526	2493	2499	2499	2499	2490	2502	2496	2505	2499	2499	2697	1176|1497	2760	2553	1497|1523	1176|1497	2697	2706	2679	2712
157	gp44 clamp loader subunit	942	879	942	942	942	1008	948	942	942	942	942	942	951	948	945	942	960	960	966	957	966	960	960	999	975	963
153	gp45 sliding clamp DNA polymerase accessory protein	666	669	669	669	660	660	666	669	666	669	666	654	693	726	660	660	687	672	678	666	672	672	687	642	687	687
143+1865 + 2162	gp46 recombination endonuclease subunit	1713	1719	1722	1722	1722	1722	1731	1722	1722	1722	1731	1722	1713	1725	1722	1722	1683	1713	2319	2238	1713	1713	1689	1704	1683	1689
141+1024	gp47 recombination endonuclease subunit	1041	1032	1047	1047	1035	1035	1050	1023	1035	744|432	954	1044	1050	1044	1035	1035	1020	1068	1029	1041	1068	1068	1020	1020	1026	1020
333+7+1152+ 1620+2019	gp48 baseplate tail tube cap	1161	1071	1326	1332	1041	885	999	1014	1116	762	1140	960	1317	2013	1044	1095	1095	1029	1077	1140	1029	1029	1095	1086	1059	1110
6	gp53 base plate wedge component	726	972	618	303	960	924	663	894	927	900	660	969	669	756	927	894	591	540	567	579	540	540	591	555	555	576
140	gp55 Sigma factor for late transcription	480	477	471	471	474	534	495	468	501	537	495	474	537	480	471	471	558	519	522	513	519	519	558	516	534	558
202	gp61 DNA primase subunit	972	834	999	999	993	969	990	987	825	987	990	996	972	813	993	978	1029	1005	1035	1059	1005	1005	1029	1029	1029	1023
167	gp62 clamp loader subunit	333	405	381	390	402	405	387	405	402	405	387	171	369	384	402	405	564	579	582	489	573	579	564	573	579	564
325+1472	DexA exonuclease A	690	684	591	384	678	684	690	678	684	684	678	672	684	687	678	681	684	666	681	693	666	666	684	675	669	678
203	NrdA ribonucleotide reductase A subunit	2328	2307	2301	2301	2319	2304	2331	2304	2310	2313	2325	2298	2298	2301	2298	2310	2265	1713	1422	2226	1713	1713	2265	2256	2244	2256
311+1531+ 1776	NrdC glutaredoxin	249	177	276	261	237	237|240	246|249	177	240	270|237	246	237|243	327|237	279|249	243	216|237	264	228	273	300	231	228	264	273	282	279
168	RegA translational repressor of early genes	444	420	423	423	426	429	426	450	432	426	426	456	438	462	429	429	369	357	369	381	357	357	369	369	363	378
318+1660 + 2793	Td thymidilate synthase	636	633	660	678	708	810	642	711	645	729	687	711	702	714	702	711	1878	840	834	903	840	840	861	858	1233	861
138	UvsW RNA-DNA + DNA-DNA helicase	1464	1464	663|840	1512	1473	1473	1464	1476	1473	1467	1452	1464	1482	1479	1473	1488	1764	1482	1512	1524	1488	1482	1512	1500	1503	1515
204+1777	NrdB ribonucleotide reductase B subunit	1155	1164	1188	1185	1167	1167	1185	1164	1167	978	1188	1161	1194	1167	1167	1161	1765	972	1131	1125	999	972	1179	1176	1161	1173

*** NOTE: gp51 (25/26), uvsX, uvsY (each in 23/26) and gp59 (22/26) are nearly universal among T4-like phages

Suppl. Table 3: Non-cyano T4-like "core" beyond the T4-core. Numbers listed for each phage are as in Suppl. Table 2.

count	T4-GC #	Function	T4	44RR	Aeh1	KVP40	PHG25	PHG31	RB32	RB43	RB49	RB69	missing in which cyanophages?
1	1642	dCMP deaminase	582	519	549	453	519	519	582	525	507	510	all
2	1475	Dda DNA helicase	1320	1320	1365	1236	1320	1320	1320	1332	1392	1314	all
3	1668	DsbA dsDNA binding protein, late transcription	270	288	288	273	285	288	270	264	276	288	all
4	1586	gp1 dNMP kinase	726	675	690	639	684	675	726	663	657	735	all
5	1593+2021+107 +1204+1328+39 7+1122+511+50	gp7 baseplate wedge initiator	3099	3060	3492	3498	3057	3060	3099	3084	3087	3099	P-RSM4, P-SSM7, S-PM2, Syn1, Syn9, Syn19, Syn33, S-SSM5, S-SM1
6	398 + 1595	gp9 baseplate wedge tail fiber connector	867	858	927	984	858	858	867	864	855	864	P-SSM2, P-HM1, P-HM2, S-PM2, Syn1, S-SM2
7	1596+2377+2022	gp10 baseplate wedge subunit and tail pin	1809	1815	2157	2247	1815	1815	1806	1818	1803	1815	all
8	1597+2378	gp11 base plate wedge component	660	663	975	705	663	663	660	663	645	660	all
9	1796+1598+2379	gp12 short tail fiber	1584	1401	1311	1422|1539	1398	1401	1551	1392	1401	1551	all
10	1606	gp24 precursor of head vertex subunit	1284	1233	1179	897	1233	1233	1284	1323	1242	1284	all
11	1627	gp30 DNA ligase	1464	1506	1488	1344	1515	1506	1461	1524	1497	1494	all
12	1639	gp31 head assembly cochaperone with GroEL	336	339	411	339	312	339	336	345	324	333	all
13	1669	gp34 long tail fiber, proximal subunit	3870	3669	3711	3771	3666	3669	3870	3660	3741	3834	all
14	1525	gp49 recombination endonuclease VII	474	474	486	456	474	474	474	483	474	474	all
15	1684	gp52 DNA topoisomerase subunit	1329	1668	1317	1287	1635	1665	1329	1164	1365	1326	all
16	1621	gp54 baseplate tail tube initiator	963	858	996	747	858	858	966	864	933	963	all
17	3	gp59 loader of gp41 DNA helicase	654	660	648	651	660	660	654	666	648	528	S-PM2, Syn1, S-SSM7, P-SSM7
18	1464	gp60+39 DNA topoisomerase subunit	1551	1824	1842	1794	1824	1824	1818	1893	1824	1821	all
19	1491	Hypothetical-Protein	165	195	468	477	189	186	165	501	177	279	all
20	1584	Hypothetical with 5' RNA ligase family domain	459	429	495	456	438	429	456	624	465	456	all
21	1630	Hypothetical with DUF1768 domain	459	450	465	465	423	450	459	441	480	414	all
22	1542+2721	NrdC.11 hypothetical protein	1011	975	954	1041	975	975	1014	987	699	990	all
23	1523	NrdD anaerobic NTP reductase large subunit	2851	1827	2115	1836	1827	1827	1818	2124	1863	1818	all
24	1519+2154	NrdH glutaredoxin	309	276	285	240	276	276	309	279	270	273	all
25	1648	PseT polynucleotide 5'-kinase and 3'-phosphatase	906	888	918	918	900	888	909	888	879	900	all
26	1698	RIIA-RIIB membrane-associated	939	1134	1317	1038	942	1134	939	3150	993	936	all
27	870	RNaseH ribonuclease	918	924	921	933	915	924	918	936	948	873	all but S-PM2, Syn1
28	1653	RnlA RNA ligase	1125	1152	1170	1146	1152	1152	1125	1113	1170	1125	all
29	1553	Tk thymidine kinase	582	576	600	585	573	576	582	582	597	582	all
30	1557	Tk.4 hypothetical protein	468	489	609	513	486	489	468	399	456	465	all
31	1559	Vs.1 hypothetical with transglycosylase SLT domain	546	552	525	627	534	552	546	636	591	543	all
32	1599 + 2023	Wac fibritin neck whiskers	1464	1764	3108	1680	1761	1764	1458	2289	1770	1443	all

Suppl. Table 4: Cyano T4-like core genes ***. Numbers listed for each phage are as in Suppl. Table 2.

			Prochlorococcus phages						*Synechococcus* phages									
Count	T4-GC #	GENE DESCRIPTION	P-SSM2	P-SSM4	P-HM1	P-HM2	P-RSM4	P-SSM7	S-PM2	Syn9	Syn19	Syn33	Syn1	S-ShM2	S-SM2	S-SSM7	S-SSM5	S-SM1
1	280	PsbA photosystem II D1 protein	1083	1098	1113	1089	1089	1167	1292	1137	1080	1080	1080	1077	909	1185	1077	1080
2	184	MazG pyrophosphatase	417	402	405	405	402	417	408	402	402	402	408	402	474	426	402	402
3	322	PhoH P-starvation inducible protein	753	774	747	762	762	771	753	765	762	762	756	768	756	768	762	762
4	170	Hsp20 small heat shock protein	459	450	471	498	450	441	411	483	507	492	462	501	492	432	507	447
5	267	Hli03 high-light inducible protein	114|108|219|144	111|207|108	108|114|222	108|114|222	201|165|114	210|147|114	120|195	204|135	255|153	108|219	210|120	210	204	213|156	108|219	255|147
6	150	CobS porphyrin biosynthetic protein	1095	1095	1065	1065	1104	1092	1119	1071	1080	1074	1098	1059	1146	1116	1074	1086
7	15	Virion structural protein	1263	1341	1248	1251	1431	1380	1395	1404	1362	1374	1398	1809	1443	1413	1401	1386
8	4	Hyp. with DUF1825 domain	333	333	321	324	333	330	348	336	327	351	321	336	345	294	333	369
9	146	Hyp. with carboxypeptidase domain	2214	2235	2226	2226	2223	2151	2223	2124	2160	2112	2136	2169	2124	2256	2184	2109
10	190	Hyp. with CTP transerase domain	1275	1152	570	552	1197	1197	1224	1149	1197	1158	570	1179	1227	1383	1197	1200
11	139	Hyp. with Methylamine utilization domain	414	441	429	429	426	444	399	438	438	420	396	447	414	408	432	432
12	101+155	Hyp. with Phytanoyl-CoA-dioxygenase domain	588|642|591|585|567|612|585|498	540	627|588	612	612|558	582	597	585|624	564|633	549	600|387|513	660|648	552	645|648|597	615|555	660|555|540
13	312+443+1092+1149	Hypothetical protein	411	240	237	231	237	246	237	237	234	237	237	243	225	234	234	234
14	176	Hypothetical protein	342	381	426|393	393|423	384	456	468	336	375	351	507	378	396	336	474	381
15	201	Hypothetical protein	294	402	417	417	393	393	495	429	429	381	435	414	402	426	393	429
16	313	Hypothetical protein	624	465	456	456	468	465	435	462	471	468	435	465	483	576	399	471
17	321	Hypothetical protein	327|282	177	243	243	231	231	240	231	243	240	249	231	246	270	231	231
18	49	Hypothetical protein	300	294	300	300	318	300	345	315	315	330	315	315	318	300	312	315
19	71	Hypothetical protein	813	927	1206	1221	996	930	579	963	999	897	1206	921	909	972	891	975
20	112+1330	Hypothetical protein	204	222	222	219	228	258	204	237	246	177	195	237	222	210	228	243
21	142	Hypothetical protein	279	273	285	261	315	249	276	252	261	249	273	252	270	288	261	255
22	152	Hypothetical protein	663	279	411	411	294	312	287	285	279	282	288	291	420	924	279	276
23	250	Hypothetical protein	240	231	291	294	303	249	240	240	243	261	342	243	237	294	297	294
24	198	Hypothetical protein	372	363	372	372	363	363	357	363	363	363	357	363	393	372	363	363
25	43	Hypothetical protein	303	192	276	276	279	171	204	186	174	186	183	174	198	195	180	180

*** NOTE: 9 genes, including 6 hypotheticals (including one with a DUF680 domain), an endonuclease, CP12 and *talC,* are nearly universal cyanophage core genes (missing only in in S-PM2)

Suppl. Table 5: Proteins that are unique to either P-HM1 or P-HM2 phage genome in pairwise comparison of these two co-isolated phages

T4-GC#	***Functional description***	***Genome location***
Unique to P-HM1		
T4-GC171	PurM	156328-156987
T4-GC404	PTOX	173106-173609
T4-GC452	peptidase M15B and M15C	9557-11437
T4-GC277	Hypothetical protein	168032-168250
T4-GC331	Hypothetical protein	2610-3095
T4-GC448	Hypothetical protein	2293-2403
T4-GC467	Hypothetical protein	30252-30749
T4-GC495	Hypothetical protein	39852-40460
T4-GC515	Hypothetical protein	90207-91184
T4-GC516	Hypothetical protein	91187-91579
T4-GC524	Hypothetical protein	112705-112881
T4-GC526	Hypothetical protein	116718-116849
T4-GC527	Hypothetical protein	120914-121168
T4-GC528	Hypothetical protein	127117-127761
T4-GC533	Hypothetical protein	139047-140504
T4-GC543	Hypothetical protein	157906-157799
T4-GC545	Hypothetical protein	158486-159028
T4-GC550	Hypothetical protein	161740-161636
T4-GC552	Hypothetical protein	162104-161988
T4-GC553	Hypothetical protein	162225-162097
T4-GC554	Hypothetical protein	166737-166838
T4-GC558	Hypothetical protein	169174-169284
T4-GC560	Hypothetical protein	173967-174227
T4-GC561	Hypothetical protein	174224-174328
T4-GC563	Hypothetical protein	176365-176544
T4-GC566	Hypothetical protein	178336-178575
T4-GC454	Hypothetical protein	16042-17223
T4-GC461	Hypothetical protein	26834-27736
T4-GC468	Hypothetical protein	31675-31842
T4-GC469	Hypothetical protein	31832-32032
T4-GC470	Hypothetical protein	32164-32012
T4-GC473	Hypothetical protein	33188-33328
T4-GC480	Hypothetical protein	35023-35205
T4-GC483	Hypothetical protein	37113-36985
T4-GC486	Hypothetical protein	38194-38307
T4-GC488	Hypothetical protein	38631-38732
T4-GC491	Hypothetical protein	39235-39375
T4-GC493	Hypothetical protein	39559-39756
T4-GC494	Hypothetical protein	39749-39892
T4-GC499	Hypothetical protein	41175-41390
T4-GC502	Hypothetical protein	42316-42492
T4-GC213	Hypothetical protein	157848-158333

(continued on next page)

Unique to P-HM2

T4-GC588	putative restriction endonuclease	133838-134473
T4-GC568	endodeoxyribonuclease	2798-3409
T4-GC573	peptidase M15B and M15C	10357-12189
T4-GC575	Kelch repeat-containing protein	27671-28591
T4-GC432	Hypothetical protein	116970-117137
T4-GC587	Hypothetical protein	124954-125154
T4-GC419	Hypothetical protein	143644-145134
T4-GC590	Hypothetical protein	161186-161338
T4-GC591	Hypothetical protein	161341-161601
T4-GC592	Hypothetical protein	168030-168140
T4-GC593	Hypothetical protein	168739-168846
T4-GC594	Hypothetical protein	169141-169257
T4-GC595	Hypothetical protein	171570-171722
T4-GC596	Hypothetical protein	176033-176221
T4-GC597	Hypothetical protein	176384-176512
T4-GC598	Hypothetical protein	176496-176606
T4-GC599	Hypothetical protein	178664-178870
T4-GC355	Hypothetical protein	178936-179310
T4-GC600	Hypothetical protein	182992-183117
T4-GC574	Hypothetical protein	19368-19865
T4-GC567	Hypothetical protein	2410-2718
T4-GC576	Hypothetical protein	33484-33230
T4-GC577	Hypothetical protein	34793-35071
T4-GC578	Hypothetical protein	35501-35602
T4-GC579	Hypothetical protein	36219-36338
T4-GC580	Hypothetical protein	39242-39370
T4-GC581	Hypothetical protein	39679-39795
T4-GC582	Hypothetical protein	40493-40708
T4-GC583	Hypothetical protein	41516-41644
T4-GC584	Hypothetical protein	42554-42727
T4-GC569	Hypothetical protein	4849-4953
T4-GC570	Hypothetical protein	4919-5023
T4-GC571	Hypothetical protein	6106-5957
T4-GC572	Hypothetical protein	6158-6289
T4-GC351	Hypothetical protein	90401-91570
T4-GC352	Hypothetical protein	91573-92319
T4-GC353	Hypothetical protein	92333-93100
T4-GC354	Hypothetical protein	93054-94793
T4-GC585	Hypothetical protein	94888-95793
T4-GC586	Hypothetical protein	95790-96236

Suppl. Table 6: *Synechococcus* phage enriched proteins. Numbers listed for each phage are as in Suppl. Table 2.

		Prochlorococcus						*Synechecoccus*									
T4-GC #	GENE DESCRIPTION	P-SSM2	P-SSM4	P-HM1	P-HM2	P-RSM4	P-SSM7	S-PM2	Syn9	Syn19	Syn33	Syn1	S-ShM2	S-SM2	S-SSM7	S-SSM5	S-SM1
881	Hypothetical protein	--	--	--	--	--	--	--	207	--	204	150	--	--	--	--	213
937	Hypothetical protein	--	--	--	--	--	--	--	165	--	168	177	168	279	--	--	--
957	Hypothetical protein	--	--	--	--	--	--	--	--	198	231	--	--	--	--	198	168
810	Hypothetical protein	--	--	--	--	--	--	204	219	--	--	--	--	231	--	--	--
927	Hypothetical protein	--	--	--	--	--	--	--	201	--	222	--	222	--	--	--	--
924	Hypothetical protein	--	--	--	--	--	--	--	165	165	168	--	--	--	--	--	162
931	Hypothetical protein	--	--	--	--	--	--	--	207	--	207	198	225	--	--	--	--
1011	Hypothetical protein	--	--	--	--	--	--	--	--	--	204	--	201	210	--	--	--
838	Hypothetical protein	--	--	--	--	--	--	219	273	339	237	279	270	297	--	--	357
1013	Hypothetical protein	--	--	--	--	--	--	--	--	--	279	243	423	--	--	--	--
730	Hypothetical protein	--	--	--	--	--	--	273	--	--	--	276	--	270	--	--	--
744	Hypothetical protein	--	--	--	--	--	--	288	237	219	297	--	270	342	--	--	213
751	Hypothetical protein	--	--	--	--	--	--	306	--	--	357	231	--	--	--	--	--
942	Hypothetical protein	--	--	--	--	--	--	--	588	591	573	690	483	573	--	--	--
945	Hypothetical protein	--	--	--	--	--	--	--	654	669	678	672	690	753	672	678	678
920	6PGDH = gnd	--	--	--	--	--	--	--	1038	1038	--	1038	1023	1038	--	1041	1041
921+1021	G6PDH = zwf	--	--	--	--	--	--	--	1446	1440	276	303	306	1440	--	1443	1437
1035	SAICAR synthetase - purine synthesis	--	--	--	--	--	--	--	--	--	699	699	699	--	--	--	--
969	virion structural protein	--	--	--	--	--	--	--	--	19452	--	--	--	--	--	18516	18543
1038	Hyp. w/ PA14 carbohydrate binding domain	--	--	--	--	--	--	--	--	--	2214	--	--	4275	--	2127	2109
928	Hyp. W/ DUF1583 domain	--	--	--	--	--	--	--	231	--	234	--	255	--	--	--	237
876	Hypothetical protein	--	--	--	--	--	--	--	183	237	201	201	210	183	195	--	219
884	Hypothetical protein	--	--	--	--	--	--	--	222	162	159	150	--	165	--	159	165
922	Hypothetical protein	--	--	--	--	--	--	--	180	183	153	162	--	159	--	--	204
987	Hypothetical protein	--	--	--	--	--	--	--	--	219	--	222	228	189	--	219	234
988	Hypothetical protein	--	--	--	--	--	--	--	--	249	--	--	273	225	--	240	240
900	Hypothetical protein	--	--	--	--	--	--	--	189	240	252	--	231	--	--	--	--
903	Hypothetical protein	--	--	--	--	--	--	--	168	--	159	--	--	--	--	168	189
919	Hypothetical protein	--	--	--	--	--	--	--	129	219	--	--	--	120	--	--	171
923	Hypothetical protein	--	--	--	--	--	--	--	135	--	--	--	252	225	--	--	135
934	Hypothetical protein	--	--	--	--	--	--	--	585	585	621	--	627	--	--	--	--
943	Hypothetical protein	--	--	--	--	--	--	--	351	--	--	--	--	399	--	399	477
948	Hypothetical protein	--	--	--	--	--	--	--	180	--	180	177	219	--	--	--	--
1010	Hypothetical protein	--	--	--	--	--	--	--	--	--	135	144	144	--	--	--	--
1021	Hypothetical protein	--	--	--	--	--	--	--	--	--	276	303	306	--	--	--	--
1026	Hypothetical protein	--	--	--	--	--	--	--	--	--	300	396	429	--	--	--	--
1036	Hypothetical protein	--	--	--	--	--	--	--	--	--	273	273	270	--	--	--	--
755	Hypothetical protein	--	--	--	--	--	--	396	--	--	--	--		--	--	357	363
835	Hypothetical protein	--	--	--	--	--	--	543	546	--	--	--	546	--	--	--	
901	Hypothetical protein	--	--	--	--	--	--	--	195	198	--	--		--	--	--	216
918	Hypothetical protein	--	--	--	--	--	--	--	240	--	216	--	225	--	--	--	--
930	Hypothetical protein	--	--	--	--	--	--	--	120	--	309	--	189	--	--	--	--
953	Hypothetical protein	--	--	--	--	--	--	--	--	204	--	--	--	--	--	216	180
955	Hypothetical protein	--	--	--	--	--	--	--	--	264	--	--	--	--	--	285	264
956	Hypothetical protein	--	--	--	--	--	--	--	--	159	--	--	--	--	--	141	141
958	Hypothetical protein	--	--	--	--	--	--	--	--	177	--	--	--	--	--	177	177
959	Hypothetical protein	--	--	--	--	--	--	--	--	339	--	--	--	--	--	231	222
964	Hypothetical protein	--	--	--	--	--	--	--	--	138	--	--	--	--	--	138	264

Suppl. Table 7: *Prochlorococcus* phage enriched proteins. Numbers listed for each phage are as in Suppl. Table 2.

T4-GC #	GENE DESCRIPTION	P-SSM2	P-SSM4	P-HM1	P-HM2	P-RSM4	P-SSM7	S-PM2	Syn9	Syn19	Syn33	Syn1	S-ShM2	S-SM2	S-SSM7	S-SSM5	S-SM1
163	***Possible PsbN photosystem protein****	303	225	210	210	150	306	--	--	--	--	--	--	--	--	--	--
436	Hli04_PSSM4	--	135	135	201	219	--	--	--	--	--	--	--	--	--	--	--
413	PcyA, phycocyanobilin biosynthesis protein	--	690	--	--	729	717	--	--	--	--	--	--	--	--	--	--
285	***Hypothetical protein****	282	288	291	288	288	264	--	--	--	--	--	--	--	--	--	--
429	Hypothetical protein	--	210	204	204	207	--	--	--	--	--	--	--	--	--	--	--
437	Hypothetical protein	--	174	180	177	--	201	--	--	--	--	--	--	--	--	--	--
485	Hypothetical protein	--	--	177	177	183	204	--	--	--	--	--	--	--	--	--	--
95	Hypothetical protein	252	339	--	--	252	201	--	--	--	--	--	--	--	--	--	--
367	Hypothetical protein	--	159	207	186	--	--	--	--	--	--	--	--	--	--	--	--
387	Hypothetical protein	--	234	249	249	--	--	--	--	--	--	--	--	--	--	--	--
391	Hypothetical protein	--	174	177	177	--	--	--	--	--	--	--	--	--	--	--	--
423	Hypothetical protein	--	216	219	222	--	--	--	--	--	--	--	--	--	--	--	--
466	Hypothetical protein	--	--	264	264	348	--	--	--	--	--	--	--	--	--	--	--
496	Hypothetical protein	--	--	198	195	171	--	--	--	--	--	--	--	--	--	--	--
506	Hypothetical protein	--	--	261	210	201	--	--	--	--	--	--	--	--	--	--	--
542	Hypothetical protein	--	--	126	126	--	189	--	--	--	--	--	--	--	--	--	--
596	Hypothetical protein	--	--	--	189	228	210	--	--	--	--	--	--	--	--	--	--
79	Hypothetical protein	216	168	--	--	162	--	--	--	--	--	--	--	--	--	--	--
82	***Hypothetical protein***	288	225	264	249	249	234	--	--	--	--	--	--	--	297	243	--
224	***Hypothetical protein***	225	159	183	162	213	207	--	--	--	--	--	--	--	207	--	--

Suppl. Table 8: Summary of cyano T4 proteomics experiments. Comparative proteomics = experimentally determined protein content in purified virus particles to determine the structural proteins in three sequenced T4-like virus genomes. An “Y” means the protein was detected, “--“ means the protein is annotated in the genome but no peptides were detected, “NP” means the protein is not present in the genome, “counts” are the number of peptide fragments detected per protein, "copy # in T4" refers to the biochemically and ultrastructurall determined copy number ofproteins in the coliphage T4 particle. Ten of these proteins, in italics, have similar distributions among 9 cyanophages and may be functionally linked.

		Proteomic data						Genomic distribution of the genes, sizes in nucleotides															
		S-SM1[1]		S-PM2[2]		SYN9[3]																	
T4-GC #	DEFINITION	DETECTED	COUNTS	DETECTED	COUNTS	DETECTED	COPY # in T4	PSSM2	PSSM4	P-HM1	P-HM2	P-RSM4	P-SSM7	S-PM2	SYN9	Syn19	Syn33	Syn1	S-ShM2	S-SM2	S-SSM7	S-SSM5	S-SM1
125	gp18	Y	70	Y	18	Y	138	2190	2250	2010	2034	2253	2250	2232	2262	2259	2253	1908	1413	2247	2421	2253	2250
126	gp19	Y	73	Y	3	Y	144	588	591	615	615	585	588	615	609	558	579	618	708	612	684	585	588
127	gp20	Y	18	Y	5	Y	12	1677	1614	1671	1683	1602	1614	1695	1602	1596	1335	1680	1650	1668	1692	1605	1602
131	gp23	Y	119	Y	22	Y	960	1413	1389	1368	1368	1398	1389	1407	1374	1374	1380	1407	1398	1404	1407	1395	1377
106	gp6	Y	25	Y	3	Y	12	1944	1989	1851	1851	2028	2031	1809	2031	2025	2031	1809	2133	2112	1875	2028	2019
116	gp13	Y	13	Y	5	-	10	846	807	816	816	810	807	831	807	807	807	831	807	846	819	807	807
118	gp15	Y	15	Y	5	-	6	843	1032	1005	1005	786	789	801	789	1032	786	801	837	1002	1083	786	786
133	gp3	Y	18	Y	2	-	6	534	552	558	546	549	549	510	573	570	549	506	567	576	561	549	549
408	gp22	Y	22	Y	3	-	115	1101	1002	1053	1053	1047	1050	1179	1038	1026	1041	1185	1131	1098	1089	1038	1020
333	gp48	Y	13	Y	2	-	6	1161	1071	1326	1332	1041	885	999	1014	1116	762	1140	960	1317	2013	1044	1095
108	gp8	Y	21	Y	19	Y	12	1602	1533	1515	1515	1539	1533	1905	1533	1533	1533	1884	1533	1551	1596	1533	1533
402	*structural protein*	*Y*	*14*	*NP*	-	*Y*	*NP*	--	*7974*	--	--	*6711*	*6543*	--	*5340*	*6489*	*5724*	--	*6687*	--	--	*5949*	*5967*
346	*fiber*	*Y*	*16*	*NP*	-	*Y*	*NP*	--	*3957*	--	--	*3963*	*3966*	--	*3954*	*3978*	*3285*	--	*3816*	--	--	*3963*	*3963*
344	*Structural protein*	*Y*	*16*	*NP*	-	*Y*	*NP*	--	*1323*	--	--	*1326*	*1323*	--	*1329*	*1323*	*1326*	--	*1320*	--	--	*1326*	*1326*
398	*gp9*	*Y*	*25*	*NP*	-	*NP*	*18*	--	*1230*	--	--	*1236*	*1227*	--	*1221*	*1239*	*1221*	--	*945*	--	*735*	*1227*	*1227*
403	*Structural protein*	*Y*	*60*	*NP*	-	*NP*	*NP*	--	*534*	--	--	*537*	*540*	--	--	*531*	*537*	--	--	--	--	*534*	*537*
334	*Structural protein*	*Y*	*34*	*NP*	-	-	*NP*	--	*990*	--	--	*795*	*837*	--	*846*	*810*	*846*	--	*606*	--	--	--	*795*
347	*Structural protein*	*Y*	*32*	*NP*	-	-	*NP*	--	*417*	--	--	*417*	*441*	--	*918*	*417*	*417*	--	*435*	--	--	*417*	*423*
399	*Structural protein*	*Y*	*8*	*NP*	-	-	*NP*	--	*4332*	--	--	*4143*	*4128*	--	*4161*	*4200*	--	--	*3555*	--	--	*4161*	*4164*
426	*Structural protein*	*Y*	*17*	*NP*	-	-	*NP*	--	*540*	--	--	--	*582*	--	*525*	*558*	*558*	--	*861*	--	--	*513*	*594*
345	*Structural protein*	-	-	-	-	*Y*	*NP*	--	*1485*	--	--	*1452*	*1455*	--	*1506*	*1491*	*1488*	--	*1479*	--	--	*1449*	*1485*
512	Structural protein	NP	-	Y	2	NP	NP	--	--	534	534	--	--	507	--	--	--	513	--	--	--	--	--
537	Structural protein	NP	-	Y	5	NP	NP	--	--	864	864	--	--	951	--	--	--	858	--	--	--	--	--
425	S-layer domain	Y	17	NP	-	NP	NP	--	573	--	--	573	633	--	--	--	--	--	--	--	564	573	573
400	Structural protein	Y	17	NP	-	Y	NP	--	7248	6654	6669	7467	7605	--	7176	7293	7209	--	7257	7560	--	7458	7173
15	Structural protein	Y	12	-	-	Y	NP	1263	1410	1248	1251	1431	1380	1395	1404	1362	1374	1398	1809	1443	1413	1401	1386
6	gp53	Y	6	-	-	-	6	726	972	618	303	960	924	663	894	927	900	660	969	669	756	927	894
190	Cytidyldyltransfera	Y	25	-	-	-	NP	1275	1152	570	552	1197	1197	1224	1149	1197	1158	570	1179	1227	1383	1197	1200
117	gp14	Y	5	-	-	-	5	1413	927	1329	1329	933	927	879	1173	921	1173	879	1164	1455	2289	927	927
129	gp21	Y	8	-	-	-	3	651	645	648	648	645	645	645	645	723	645	645	645	651	735	645	645
105	gp25	Y	15	-	-	-	6	402	420	390	396	420	420	393	417	420	420	393	438	402	417	420	420
9	gp4	Y	4	-	-	-	??	438	426	435	462	426	420	438	420	420	420	342	438	444	480	426	474
340	gp5	Y	11	-	-	-	3	2259	2310	870	873	2541	2553	2946	2508	2385	2580	2922	2484	1848	3027	2544	2553
119	Structural protein	Y	13	NP	-	-	NP	816	1386	702	894	1098	519	--	1332	1164	1371	276	480	2127	2985	1206	1488
607	Lysozyme murein	Y	16	NP	-	NP	NP	--	--	--	--	2802	--	--	--	--	--	--	--	--	--	2862	2757
1038	PA14 domain	Y	5	NP	-	NP	NP	--	--	--	--	--	--	--	--	--	2214	--	--	4275	--	2127	2109
1426	Structural protein	Y	17	NP	-	NP	NP	--	--	--	--	--	--	--	--	--	--	--	--	--	--	--	2757
1428	Structural protein	Y	25	NP	-	NP	NP	--	--	--	--	--	--	--	--	--	--	--	--	--	--	--	948
1453	Structural protein	Y	12	NP	-	NP	NP	--	--	--	--	--	--	--	--	--	--	--	--	--	--	--	834
1454	Structural protein	Y	10	NP	-	NP	NP	--	--	--	--	--	--	--	--	--	--	--	--	--	--	--	1422
1455	Structural protein	Y	7	NP	-	NP	NP	--	--	--	--	--	--	--	--	--	--	--	--	--	--	--	489
641	Structural protein	Y	9	NP	-	NP	NP	--	--	--	--	2640	--	--	--	--	2553	--	--	--	--	2589	2592
642	Structural protein	Y	5	NP	-	-	NP	--	--	--	--	609	582	--	522	--	--	--	522	--	--	582	552
969	Structural protein	Y	8	NP	-	NP	NP	--	--	--	--	--	--	--	--	19452	--	--	--	--	--	18516	18543
735	Structural protein	NP	-	Y	2	NP	NP	--	--	--	--	--	--	1350	--	--	--	1704	--	--	--	--	--
737	Structural protein	NP	-	Y	3	NP	NP	--	--	--	--	--	--	528	--	--	--	531	--	--	--	--	--
827	Structural protein	NP	-	Y	3	NP	NP	--	--	--	--	--	--	1704	--	--	--	1851	--	--	--	--	--
739	Structural protein	NP	-	Y	3	NP	NP	--	--	--	--	--	--	1749	--	--	--	--	--	--	--	--	--
829	Structural protein	NP	-	Y	9	NP	NP	--	--	--	--	--	--	3756	--	--	--	--	--	--	--	--	--
831	Structural protein	NP	-	Y	5	NP	NP	--	--	--	--	--	--	921	--	--	--	--	--	--	--	--	--
832	Structural protein	NP	-	Y	2	NP	NP	--	--	--	--	--	--	984	--	--	--	--	--	--	--	--	--
833	Structural protein	NP	-	Y	2	NP	NP	--	--	--	--	--	--	1140	--	--	--	--	--	--	--	--	--
864	Structural protein	NP	-	Y	5	NP	NP	--	--	--	--	--	--	3177	--	--	--	--	--	--	--	--	--
267	Putative HLIP	-	-	Y	2	-	NP	114| 108| 219| 144	111| 207| 108	108| 114| 222	108| 114| 222	201| 165| 114	210| 147| 114	120| 195	204| 135	255| 153	108| 219	210| 120	210	204	213| 156	108| 219	255| 147
